# An MHV-68 Mutator Phenotype Mutant Virus, Confirmed by CRISPR/Cas9-Mediated Gene Editing of the Viral DNA Polymerase Gene, Shows Reduced Viral Fitness

**DOI:** 10.3390/v13060985

**Published:** 2021-05-26

**Authors:** Erika Trompet, Arturo Temblador, Sarah Gillemot, Dimitrios Topalis, Robert Snoeck, Graciela Andrei

**Affiliations:** Laboratory of Virology and Chemotherapy, Rega Institute for Medical Research, KU Leuven, 3000 Leuven, Belgium; erikatrompet@hotmail.com (E.T.); arturojesus.tembladorperez@kuleuven.be (A.T.); sarah.gillemot@kuleuven.be (S.G.); dimitri.topalis@gmail.com (D.T.); robert.snoeck@kuleuven.be (R.S.)

**Keywords:** herpesviruses 1, DNA polymerase 2, drug resistance 3, mutator phenotype 4, viral fitness 5, CRISPR/Cas9 precise gene editing

## Abstract

Drug resistance studies on human γ-herpesviruses are hampered by the absence of an in vitro system that allows efficient lytic viral replication. Therefore, we employed murine γ-herpesvirus-68 (MHV-68) that efficiently replicates in vitro as a model to study the antiviral resistance of γ-herpesviruses. In this study, we investigated the mechanism of resistance to nucleoside (ganciclovir (GCV)), nucleotide (cidofovir (CDV), HPMP-5azaC, HPMPO-DAPy) and pyrophosphate (foscarnet (PFA)) analogues and the impact of these drug resistance mutations on viral fitness. Viral fitness was determined by dual infection competition assays, where MHV-68 drug-resistant viral clones competed with the wild-type virus in the absence and presence of antivirals. Using next-generation sequencing, the composition of the viral populations was determined at the time of infection and after 5 days of growth. Antiviral drug resistance selection resulted in clones harboring mutations in the viral DNA polymerase (DP), denoted Y383S^GCV^, Q827R^HPMP-5azaC^, G302W^PFA^, K442T^PFA^, G302W+K442T^PFA^, C297W^HPMPO-DAPy^ and C981Y^CDV^. Without antiviral pressure, viral clones Q827R^HPMP-5azaC^, G302W^PFA^, K442T^PFA^ and G302W+K442T^PFA^ grew equal to the wild-type virus. However, in the presence of antivirals, these mutants had a growth advantage over the wild-type virus that was moderately to very strongly correlated with antiviral resistance. The Y383S^GCV^ mutant was more fit than the wild-type virus with and without antivirals, except in the presence of brivudin. The C297W and C981Y changes were associated with a mutator phenotype and had a severely impaired viral fitness in the absence and presence of antivirals. The mutator phenotype caused by C297W in MHV-68 DP was validated by using a CRISPR/Cas9 genome editing approach.

## 1. Introduction

The human γ-herpesviruses Epstein–Barr virus (EBV) and Kaposi’s sarcoma-associated virus (KSHV) distinguish themselves from the α-herpesviruses (herpes simplex virus 1 (HSV-1) and 2 (HSV-2), varicella-zoster virus (VZV)) and β-herpesviruses (human cytomegalovirus (HCMV), human herpesvirus 6A/6B/7 (HHV-6A/6B/7)) by their association with a large number of malignancies [1,2,3,4]. EBV is associated with the development of several lymphoproliferative disorders (Burkitt’s lymphoma, Hodgkin lymphoma, diffuse large B-cell lymphoma and lymphoproliferative disease in immunocompromised hosts) and carcinomas of epithelial origin (nasopharyngeal carcinoma and gastric carcinoma) [5]. KSHV is linked to endothelial sarcomas (Kaposi’s sarcoma) and malignancies of B-cell origin (multicentric Castleman’s disease and primary effusion lymphoma) [6].

Though EBV and KSHV oncogenesis is mostly associated with latent gene expression, recent investigations demonstrate a significant contribution of lytic replication to viral tumorigenesis [7,8]. Therefore, antiviral therapy intended at inhibiting the expression of lytic viral proteins should be beneficial for controlling the early stages of EBV-associated malignancies. The contribution of EBV lytic replication to lymphoproliferative disease has been highlighted by several studies [9,10]. The EBV immediately early (IE) proteins BZLF1 and BRLF1 promote interleukin-6 (IL-6) secretion in lytically infected cells, sustaining early lymphoproliferative disease [11] as IL-6 (a cytokine playing a key role in maintenance of immune functions, stimulation of hematopoietic cells differentiation and persistence of inflammation) is a decisive element in various epithelial and hematological malignancies. IL-6 promotes cell survival and induces signal transducer and activator of transcription 3 (STAT3), acting through paracrine and autocrine mechanisms [11]. EBV, a virus infecting both epithelial and lymphoid cells, induces IL-6 expression. Expression of cellular and viral interleukin 10 (IL-10) is induced in lytically infected EBV cells, resulting in a more efficient growth of B-cells [12]. The involvement of cellular IL10 and viral IL-6 in the growth, survival and spread of KSHV-associated PEL, MCD and KS was also established [13,14]. Vascular endothelial growth factor (VEGF), which contributes to angiogenesis in both B-cell and epithelial malignancies, is also increased following EBV infection [15]. The lytic cycle is important for KSHV-mediated disease development. For instance, KSHV viral load in peripheral blood mononuclear cells is correlated with KS progression [16].

Some antiviral drugs that inhibit viral lytic DNA replication, such as foscarnet and ganciclovir, can repress early KS development [17]. Tumor-forming spindle cells are latently infected in most KS lesions, though a small number of cells still undergo spontaneous lytic replication. EBV is a major risk factor for the development of post-transplant lymphoproliferative disorder (PTLD), a serious and often fatal complication. Persistence of elevated viral loads or a high level of EBV DNA in the blood of transplant recipients is associated with an increased risk of PTLD [18,19]. Although antiviral therapy is not useful in PTLD due to viral latency, antiviral drug prophylaxis is able to reduce its incidence [20,21], highlighting the role of antivirals in preventing PTLD in transplant recipients. Collectively, lytic replication not only plays an indispensable function during the life cycle of γ-herpesvirus, ensuring virus propagation, but also has a fundamental role in EBV- and KSHV-associated malignancies. Additionally, lytic EBV replication contributes to infectious mononucleosis, oral hairy leukoplakia and chronic active Epstein–Barr virus (CAEBV). Hence, considering that γ-herpesvirus reactivation is an important mechanism in the pathogenesis of EBV- and KSHV-associated diseases, antivirals targeting the lytic stage should be helpful for controlling the early stages of γ-associated malignancies. The anti-γ-herpesvirus activity of known antivirals is directed toward the impediment of viral replication; no antiviral is effective on the latent phase of infection, showing limited success clinically, and none of them have been licensed for treatment of EBV or KSHV infections [22,23,24].

In vitro, a number of nucleosides (acyclovir (ACV), ganciclovir (GCV), brivudin (BVDU)), nucleotides (cidofovir (CDV), HPMP-5azaC and HPMPO-DAPy) and pyrophosphate analogues (foscarnet (PFA)) inhibit γ-herpesvirus replication [25,26,27,28,29]. However, to date, no antiviral agent has been approved for the prevention or treatment of human γ-herpesvirus. The impact of long-term antiviral treatment on EBV and KSHV disease initiation and progression has been investigated in multiple clinical trials [17,20,30,31]. The goal was to evaluate the pre-emptive effect of antivirals on the development of lymphoproliferative malignancies, i.e., GCV prophylaxis on EBV-positive PTLD development [30,32], and GCV or PFA prophylaxis on AIDS-associated KS [17]. In these studies, the most used antivirals were ganciclovir and its oral prodrug valganciclovir, and no cases of drug resistance have been reported yet [17,22,23]. However, nearly all these studies evaluated antivirals in an immunocompromised setting, a condition known to favor the emergence of drug resistance.

Nucleoside, nucleotide and pyrophosphate analogues target the viral DNA polymerase (DP) but differ in their mode of activation. In γ-herpesviruses, purine and pyrimidine nucleosides require a first phosphorylation step by a viral protein kinase (PK) or thymidine kinase (TK), respectively, and are subsequently phosphorylated by cellular kinases. The active triphosphates can then serve as a substrate of the viral DP reaction. Nucleotide analogues do not rely on viral proteins for their activation and are phosphorylated by cellular kinases to the active diphosphate form. After phosphorylation, the active metabolites compete with the natural nucleotides for incorporation into the cellular and viral DNA. Viral DNA polymerases preferentially incorporate the active form of the nucleotide analogues into viral DNA, leading to selective inhibition of viral DNA synthesis. Pyrophosphate analogues interact with the pyrophosphate-binding site of the DNA polymerase and inhibit the cleavage of the pyrophosphate moiety from deoxynucleotide triphosphates (dNTPs) [25,33,34,35]. In γ-herpesviruses, antiviral resistance to nucleoside analogues can arise at the level of the PK and/or DP (purine analogues) or at the TK and/or DP (pyrimidine analogues). For nucleotide and pyrophosphate analogues, resistance is associated exclusively with changes in the viral DP [27,36,37].

At present, there is limited knowledge regarding human γ-herpesvirus drug resistance. This can be attributed to the lack of in vitro cell culture systems permissive for efficient lytic replication [38] which impedes the isolation and characterization of drug-resistant viral variants. To overcome this hurdle, we used murine γ-herpesvirus-68 (MHV-68) in earlier investigations as a surrogate for the study of human γ-herpesviruses [39,40]. MHV-68, in contrast to EBV and KSHV, can infect and efficiently replicate in a broad range of cell types [41], allowing for selection of drug-resistant mutants [26,27,36,37]. Despite progress in understanding the molecular mechanisms of MHV-68 drug resistance, there is currently no information regarding resistance to a number of nucleoside, nucleotide and pyrophosphate analogues. Moreover, the emergence of drug resistance raises the question of the impact of mutations on viral fitness, as alterations to genes vital to replication can diminish viral fitness. Until recently, herpesvirus viral fitness studies were performed using monoculture infection systems [42,43,44]. However, dual infection competition assays, where two viral variants are grown together and the proportions of the competing viruses are carefully measured over time, are currently the “gold standard” to measure viral fitness. Competition experiments have the advantage that they can estimate the replication capacity of two viral variants under identical conditions. While earlier investigations relied on bulk [45,46] or clonal sequencing [47] to determine the ratio of wild-type to drug-resistant viruses, we developed a next-generation sequencing (NGS) approach to accurately quantify the composition of the viral population in herpesvirus dual infection competition assays [48].

In this study, we investigated the mechanism of MHV-68 resistance to nucleoside (GCV), nucleotide (CDV, HPMP-5azaC, HPMPO-DAPy) and pyrophosphate analogues (PFA) and evaluated the impact of mutations associated with drug resistance on viral replication capacity. The viral fitness of wild-type versus drug-resistant viruses was evaluated using dual infection competition assays in the absence and presence of antivirals. The frequency of the competing viral variants was measured using the Illumina NGS platform (Miseq), which allowed identifying variants in a mixed viral population with a high sensitivity. Two amino acid changes were associated with a mutator phenotype (C297W and C981Y), and the association between the C297W amino acid change and a mutator phenotype virus was validated by using CRISPR/Cas9 genome editing.

## 2. Materials and Methods

### 2.1. Cells and Virus

Vero (ATCC-CCL81) and HEK293T (ATCC-CRL-321) cells were grown in Dulbecco’s Modified Eagle’s Medium (DMEM, ThermoFisher, Merelbeke, Belgium) supplemented with 10% fetal calf serum (FCS), 2 mM L-glutamine, 0.1 mM non-essential amino acids, 1 mM sodium pyruvate and 10 mM HEPES at 37 °C in a 5% CO_2_ humidified atmosphere. MHV-68 (G2.4) was provided by Prof A. Nash, Edinburgh, United Kingdom. MHV-68 infection was performed in Vero cells with 2% FCS DMEM.

### 2.2. Compounds

The following antivirals were used: acyclovir (ACV, [9-(2-hydroxyethoxymethyl)guanine]), GlaxoSmithKline, Stevenage, UK; ganciclovir (GCV, [9-(1,3-dihydroxy-2-propoxymethyl)guanine), Roche, Basel, Switzerland; foscarnet (PFA, [phosphonoformate sodium salt]), Sigma Chemicals, St. Louis, MO, USA; cidofovir (CDV, [(S)-1-(3-hydroxy-2-phosphonomethoxypropyl)cytosine]), Gilead Sciences, Foster City, CA, USA; brivudin (BVDU, [(E)-5-(2-bromovinyl)-2′-deoxyuridine]), Searle, UK; HDVD, 1-[(2S,4S-2-(hydroxymethyl)-1,3-dioxolan-4-yl]5-vinylpyrimidine-2,4(1H,3H)-dione, Dr. D. Chu (University of Georgia, Athens, GA, USA); KAY-2-41 [1-(2-deoxy-1-methyl-4-thio-β-Dribofuranosyl)thymine and KAH-39-139 [1-(2-deoxy-4-amino-4-thio-β-Dribofuranosyl)thymine], Showa University, Tokyo, Japan; HPMP-5azaC [(1-(S)-(3-hydroxy-2-(phosphonomethoxy)propyl)-5-azacytosine] and HPMPO-DAPy [(R)-2,4-diamino-3-hydroxy-6-[2-(phosphonomethoxy)propoxy])pyrimidine], Dr M. Krecmerova, Institute of Organic Chemistry and Biochemistry, Prague, Czech Republic.

### 2.3. Selection of Drug-Resistant MHV-68

Selection of MHV-68 mutants resistant to GCV, CDV, HPMP-5azaC, HPMPO-DAPy or PFA was performed by sequential passaging of the virus under increasing concentrations of antiviral. Resistance emerged following 21 (GCV), 15 (CDV), 12 (HPMP-5azaC), 13 (HPMPO-DAPy) and 17 (single mutant) or 22 (double mutant) (PFA) passages under antiviral pressure. The heterogeneous populations of drug-resistant viruses were plaque purified by limited dilution to obtain single viral clones.

### 2.4. Genotypic Analysis

Each drug-resistant viral clone was genotyped using Sanger sequencing. After DNA extraction (QIAmp DNA kit; Qiagen Benelux BV, Venlo, The Netherlands), the viral TK, PK and DP genes were amplified by PCR (Table S1) (FastStart high-fidelity DNA polymerase; Roche Applied Science, Mannheim, Germany). The PCR products were purified (QIAquick purification kit, Qiagen Benelux BV, Venlo, The Netherlands) and sequenced using the BigDye terminator kit version 3.1 on an ABI 3730 sequencing system (ThermoFisher, Merelbeke, Belgium) (Table S1). The sequencing results were analyzed with SeqScape 2.7 (ThermoFisher, Merelbeke, Belgium).

### 2.5. 3D Modeling

Based on the already published HSV-1 DP structure (pdb code: 2GV9), a model of MHV-68 DP was built using the protein structure homology-modelling server Swiss Model (https://swissmodel.expasy.org, accessed on 1 April 2021). The altered amino acid positions of the resistant viral clones were marked in red. The figures were constructed using Pymol Delano software, open source version 0.99rc6.

### 2.6. Drug Susceptibility Profile

MHV-68 wild-type and drug-resistant viral clones were characterized by CPE reduction assays in Vero cells as described earlier [23]. For each antiviral, the required concentration to reduce virus-induced CPE by 50% compared to the untreated control (EC_50_) was determined. For each clone, at least three independent assays were performed, and the mean EC_50_ values were calculated. The level of drug resistance was expressed as the ratio EC_50_ resistant virus/EC_50_ wild-type virus. MHV-68 viral clones were considered resistant or hypersensitive at ≥2-fold change in EC_50_.

### 2.7. Replication Capacity and Relative Fitness

The viral replication capacity of Y383S^GCV^, Q827R^HPMP-5azaC^, G302W^PFA^, K442T^PFA^ and G302W+K442T^PFA^, C297W^HPMPO-DAPy^ and C981Y^CDV^ was determined in the absence and presence of antivirals at 750 PFU/well of either the wild-type virus, a drug-resistant viral clone or a 50:50 ratio of both (375 PFU wild-type virus: 375 PFU DP mutant virus), as described earlier [48]. The replication capacity of viral clone Y383S^GCV^ was evaluated using an 80:20 ratio of Y383S^GCV^/MHV-68 wild-type (600 PFU Y383S^GCV^/150 PFU wild-type virus). The composition of the viral population was determined using the Illumina Miseq NGS platform as described earlier [48]. PCR amplification of the viral DP was performed using the following primer pair: For: 5′-CCATATAGGCTACTCTACCCTC-3′, and Rev: 5′-GTAGGTCCAGAGTGGTGTATC-3′.

After NGS and data analysis, the relative fitness was calculated using the formula: (1 + s) = exp [1/t × ln (M_t_/W_t_ × W_t0_/M_t0_)], where s is the selection coefficient and t is the time (in days). M_t_ and M_t0_ are the fractions of mutant virus initially and at the time of the measurement. W_t_ and W_t0_ are the fractions of wild-type virus initially and at the time of the measurement. An RF = 1 indicates that both viruses grew with equivalent fitness, an RF <1 indicates that the mutant virus grows less efficiently than the wild-type virus and an RF >1 indicates increased fitness of the mutant virus relative to that of the wild-type virus [48,49,50]. The mean RF values from two biological replicate infection competition assays are reported. The impact of drug resistance mutations in the viral DP on viral fitness and the impact of antiviral treatment were calculated using one-way Anova (multiple comparisons to MHV-68 wild-type with the Dunnett test (*p* ≤ 0.01)).

### 2.8. Correlation between Antiviral Resistance and Relative Fitness

The strength and direction of the relationship between antiviral drug resistance and relative fitness capacity were calculated using Spearman’s rank-order correlation test. Spearman’s correlation coefficient ρ (also signified by r_s_) measures the strength and direction of association between two ranked variables (r_s_ between 0.00 and 0.19 (very weak), 0.20 and 0.39 (weak), 0.40 and 0.59 (moderate), 0.60 and 0.79 (strong) and 0.80 and 1.0 (very strong)).

### 2.9. Investigation of the Mutation Frequency in the Viral PK, TK and DP

To determine the mutation frequency of the mutator phenotype viruses C297W^HPMPO-DAPy^ and C981YCDV, three different viral clones of each mutator were selected and expanded by three rounds of amplification. A DNA extract of each clone was obtained at passage 0 and passage 3, and the emergence of spontaneous mutations in the viral PK, TK and DP was determined by NGS as mentioned earlier [48]. The mutations arising in MHV-68 PK, TK and DP were quantified (A → T, A → G, C → A; C → T, G → T; G → A, C → A, C → T), and the intermutational distance (i.e., the distance between two mutations) was quantified. Lastly, the spontaneous mutations were aligned to HSV-1, HSV-2, VZV and HCMV (Uniprot) to determine if the corresponding amino acids were linked to known polymorphisms or mutations associated with drug resistance.

### 2.10. CRISPR/Cas9 Genome Editing of MHV-68 DP Amino Acid Position C297

#### 2.10.1. Plasmid Construct

The CRISPR/Cas9 plasmid construct was assembled using the GeneArt CRISPR Nuclease Vector, which contains an orange fluorescent protein reporter (ThermoFisher, Merelbeke, Belgium). CRISPRdirect (https://crispr.dbcls.jp/, accessed on 5 October 2015) was used to select a target-specific sgRNA with a reduced number of off-target sites, using the MHV-68 DP sequence as input (sgRNA sequence: 5′-GTCATTTGATATAGAGTGTT-3′) (Figure S1).

Target-specific oligonucleotides (For: 5′-GTCATTTGATATAGAGTGTTGTTTT-3′, and sgRNA Rev: 5′-AACACTCTATATCAAATGACCGGTG-3′) were synthesized with 3′ overhang ends compatible to clone into the GeneArt Vector. The circularized vector was transformed into One Shot TOP10 chemically competent E. coli bacteria (ThermoFisher, Merelbeke, Belgium). Ampicillin-resistant colonies were cultured overnight for DNA extraction (Wizard Plus SV Minipreps DNA Purification System, Promega, Leiden, The Netherlands). The presence of the correct insert was confirmed by Sanger sequencing, using primers flanking the cloning site of the vector (For: 5′-GAGGGCCTATTTCCCATGAT-3′, and Rev: 5′-ACCATGATTACGCCAAGCTC-3′). Following successful cloning, midipreps of 50 mL were performed using the PureLink.HiPure Plasmid Midiprep Kit (ThermoFisher, Merelbeke, Belgium). Plasmid concentration and purity were assessed with Nanodrop ND-1000 (Isogen Life Science, De Meern, The Netherlands).

#### 2.10.2. Transfection/Infection

After cloning the sgRNA coding sequence into the GeneArt Vector, the purified plasmid and the homologous recombination (HR) template were transfected into HEK293T cells by reverse transfection using Lipofectamine 3000 (ThermoFisher, Merelbeke, Belgium), following the manufacturer’s instructions. Then, 2.5 × 10^5^ transfected HEK293T cells were plated in each well of a 24-well plate. The following asymmetric template was used: AGAGATAGACTGTGGTCTGGGAACATCTGTCATCATCCAGAGCTCTCCTCCTGGCCCCCCT-ACAATATCCTGTCATTTGATATAGAGTGGTTAGGTGAATGTGGGTTTCCCTGTG-CCCTGAAAGAA, where the target nucleotide change (T to G) is indicated in bold and highlighted in gray, and a silence mutation (A, bold) was included in the PAM (underlined). Consequently, the thymine at position 891 of MHV-68 DP was altered to guanine (T891G).

The next day, following transfection, the cells were infected with 25 PFU/well, 50 PFU/well or 100 PFU/well of MHV-68 wild-type. After 2 h, the residual virus was removed, and the cells were incubated with 1 mL DMEM 2%. The supernatants were harvested 24 h, 48 h and 120 h post-infection. The obtained viral stocks were plaque purified by limited dilution. The clones were screened for the presence of the T891G nucleotide change by Sanger sequencing and NGS, as described earlier.

## 3. Results

### 3.1. MHV-68 Mutants Selected under Pressure of GCV-, HPMP-5azaC- or PFA-Harbored Mutations in the Viral DNA Polymerase (DP)

**Figure 1:**
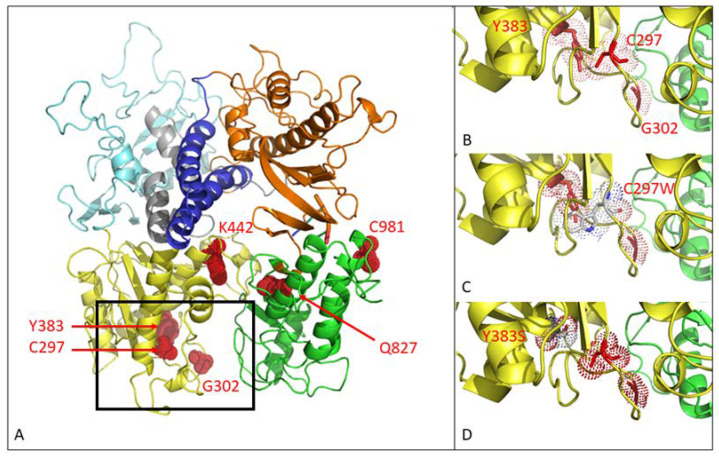
Three-dimensional modeling of mutations in the MHV-68 viral DNA polymerase. (**A**) MHV-68 DNA polymerase consists of 6 domains. Mutations C297W^HPMPO-DAPy^, G302W^PFA^, Y383S^GCV^ and K442T^PFA^ map to the 3′–5′ exonuclease domain (yellow). Q827R^HMP-5azaC^ and C981Y^CDV^ map to the thumb domain (green). There were no mutations in the other domains (5′–3′ exonuclease domain (light blue), NH2 domain (gray), palm (orange), finger (dark blue)). (**B**) Close view of positions C297, G302 and Y383. (**C**) Close view of the amino acid change C297W. (**D**) Close view of the amino acid change Y383S.

Selection with GCV, HPMP-5azaC or PFA generated five MHV-68 drug-resistant viral clones with amino acid changes in the viral DP (without changes in the PK or TK genes). The MHV-68 DP consists of six domains: 3′–5′ exonuclease domain (yellow), thumb (green), 5′–3′ exonuclease domain (light blue), NH2 domain (gray), palm (orange) and finger (dark blue) (Figure 1). Drug resistance selection with GCV and PFA gave rise to mutations in the 3′–5′ exonuclease domain. Selection with GCV led to an Y383S^GCV^ amino acid change, whereas under pressure of PFA, three MHV-68 drug-resistant viral clones were obtained: two clones with single substitutions (G302W^PFA^ and K442T^PFA^) and one clone bearing both mutations (G302W+K442T^PFA^). Selection with HPMP-5azaC resulted in the amino acid change Q827R^HPMP-5azaC^, located in the thumb domain of the viral DP.

**Figure 2:**
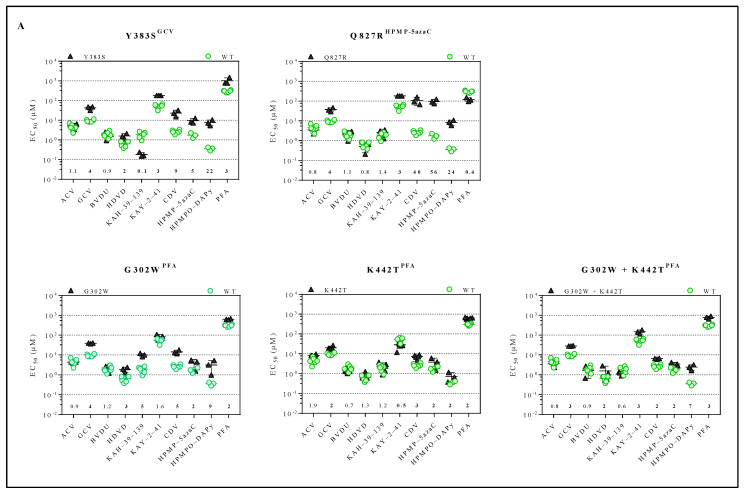

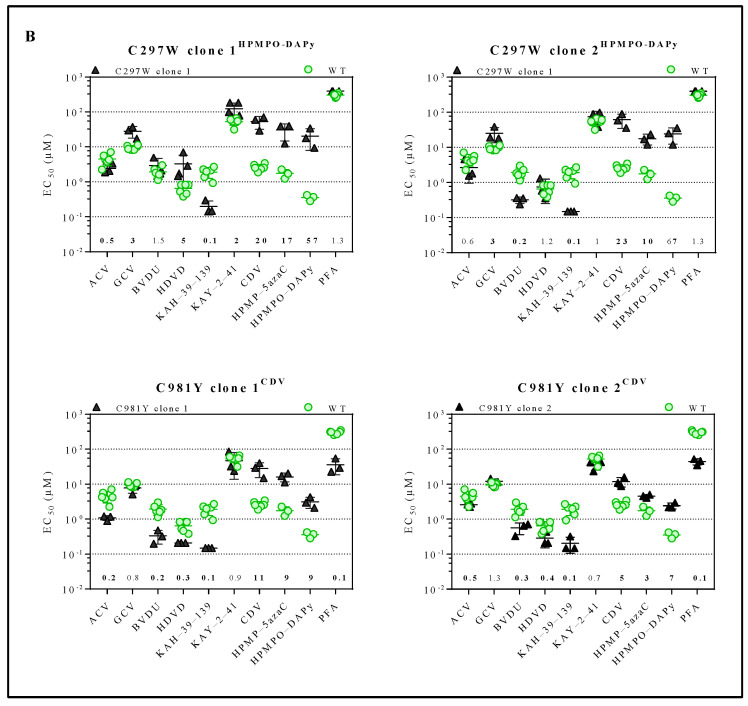
Drug susceptibility profile of MHV-68 wild-type and drug-resistant clones. (**A**) Drug susceptibility profile of viral variants Y383S^GCV,^ Q827R^HPMP-5azaC^, G302W^PFA^, K442T^PFA^ and G302W+K442T^PFA^. (**B**) Drug susceptibility profile of 2 different viral clones of mutator phenotype viruses C297W^HPMPO-DAPy^ and C981Y^CDV^. In each graph, the wild-type virus is marked with a circle (green), and the drug-resistant virus is marked with a triangle (black). The fold resistance, calculated as the EC_50_ of the drug-resistant virus to the wild-type virus, is marked at the bottom of the graph. MHV-68 viral clones were considered drug-resistant or hypersensitive at ≥2-fold change in EC_50_ (bold).

The inhibitory effects of a panel of antivirals (ACV, GCV, BVDU, HDVD, KAH-39-139, KAY-2-41, CDV, HPMP-5azaC, HPMPO-DAPy or PFA) against the drug-resistant MHV-68 viral clones were determined (Figure 2A and Table S2). The compound concentration to reduce virus-induced CPE by 50% compared to the untreated control, i.e., EC_50_, was calculated for the drug-resistant viruses. The viral variants Y383S^GCV^ and Q827R^HPMP-5azaC^ were resistant to GCV (4-fold) and HPMP-5azaC (5- and 56-fold, respectively) and were cross-resistant to KAY-2-41 (3-fold), CDV (9- and 40-fold, respectively) and HPMPO-DAPy (22- and 24-fold, respectively). The viral variant Y383S^GCV^ also demonstrated cross-resistance to HDVD (2-fold) and PFA (3-fold), and Q827R^HPMP-5azaC^ was hypersensitive to PFA (0.4-fold). The viral variants G302W^PFA^, K442T^PFA^ and G302W+K442T^PFA^ were resistant to PFA (2-, 2- and 3-fold, respectively) and had cross-resistance to GCV (4-, 2- and 3-fold, respectively), CDV (5-, 3- and 2-fold, respectively), HPMP-5azaC (2-fold) and HPMPO-DAPy (9-, 2- and 7-fold, respectively). Viral variants G302W^PFA^ and G302W+K442T^PFA^ were resistant to HDVD (3-fold), and the viral variant G302W^PFA^ was resistant to KAH-39-139 (5-fold), while the other selected clones under PFA remained equally sensitive to this drug. None of the stated mutants showed resistance to ACV or BVDU.

### 3.2. MHV-68 Mutants Selected under Pressure of CDV or HPMPO-DAPy Were Associated with a Mutator Phenotype Virus

In contrast to Y383S^GCV^ and Q827R^HPMP-5azaC^, G302W^PFA^, K442T^PFA^ and G302W+K442T^PFA^ genotyping of C297W^HPMPO-DAPy^ (Sanger sequencing) and C981Y^CDV^ (NGS) showed multiple spontaneous mutations arising throughout the viral PK, TK and DP genes, suggesting that these substitutions were associated with a mutator phenotype virus (Table S3).

The C297W^HPMPO-DAPy^ amino acid change is in the 3′–5′exonuclease domain (yellow), and C981Y^CDV^ is in the thumb domain (green) of the viral DP (Figure 1A). Amino acid changes at positions C297, G302, and Y383 are located closely together in the 3′–5′ exonuclease domain, as depicted in Figure 1B. The amino acid substitution C297W is visualized in Figure 1C, where the change of a cysteine to tryptophan appears to induce stereochemical hindrance, which may lead to an impaired function of the 3′–5′ exonuclease domain, giving rise to a mutator phenotype virus. The Y383S amino acid change is depicted in Figure 1D, where no stereochemical hindrance appears to occur.

For each mutator phenotype virus, the drug susceptibility profile of two viral clones (C297W^HPMPO-DAPy^ clones 1 and 2, and C981Y^CDV^ clones 1 and 2) was determined (Figure 2B and Table S2). Interestingly, C297W^HPMPO-DAPy^ clones 1 and 2 had different drug susceptibility profiles. C297W^HPMPO-DAPy^ clones 1 and 2 were both resistant to GCV (3-fold), CDV (20- and 23-fold, respectively), HPMP-5azaC (17- and 10-fold, respectively) and HPMPO-DAPy (57- and 67-fold, respectively) and demonstrated hypersensitivity to KAH-39-139 (0.1-fold). C297W^HPMPO-DAPy^ clone 1 remained equally sensitive to BVDU, while clone 2 was hypersensitive (0.2-fold). C297W^HPMPO-DAPy^ clone 1 was resistant to HDVD (5-fold) and KAY-2-41 (2-fold), while clone 2 remained equally sensitive. C981Y^CDV^ clones 1 and 2 had a similar drug susceptibility profile and demonstrated resistance to nucleotide analogues (CDV (11- and 5-fold, respectively), HMP-5azaC (9- and 3-fold, respectively) and HPMPO-DAPy (9- and 7-fold, respectively)). Both viral clones were hypersensitive to ACV (0.3- and 0.5-fold), BVDU (0.2- and 0.3-fold), KAH-39-139 (0.1-fold) and PFA (0.1-fold). 

### 3.3. Without Antiviral Pressure, the Viral Fitness of Q827R^HPMP-5azaC^, G302W^PFA^, K442T^PFA^ and G302W+K442T^PFA^ Remained Equal to Wild-Type Virus, While Y383SGCV Had an Increased Viral Fitness

The replication capacity of MHV-68 drug-resistant clones versus the wild-type virus was determined by dual infection competition assays. A mixture containing equal infectious units of wild-type and drug-resistant viruses was cultured for 5 days. The composition of the viral population in the absence of antivirals was determined by NGS at the time of infection and after 5 days of growth. The replication capacity of each viral clone was evaluated by calculating the 1 + *s* relative fitness (RF) values (Table S4).

Without antiviral pressure, there was no significant increase or decrease in the viral fitness of Q827R^HPMP-5azaC^ (RF = 1.08), G302W^PFA^ (RF = 0.98), K442T^PFA^ (RF = 1.02) or G302W+K442T^PFA^ (RF = 0.97) compared to the untreated wild-type virus. Interestingly, Y383S^GCV^ had an increased fitness compared to the untreated wild-type virus (RF = 1.33, *p* = 0.009).

### 3.4. Antiviral Drug Treatment Altered the Viral Fitness of Y383S^GCV^, Q827R^HPMP-5azaC^, G302W^PFA^, K442T^PFA^ and G302W+K442T^PFA^

**Figure 3:**
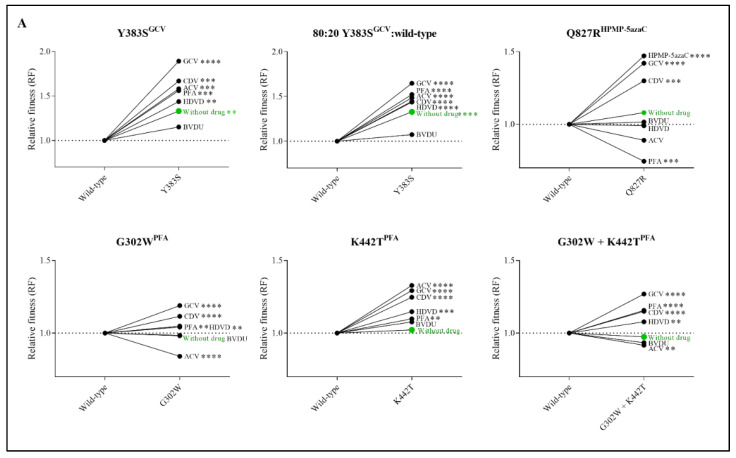

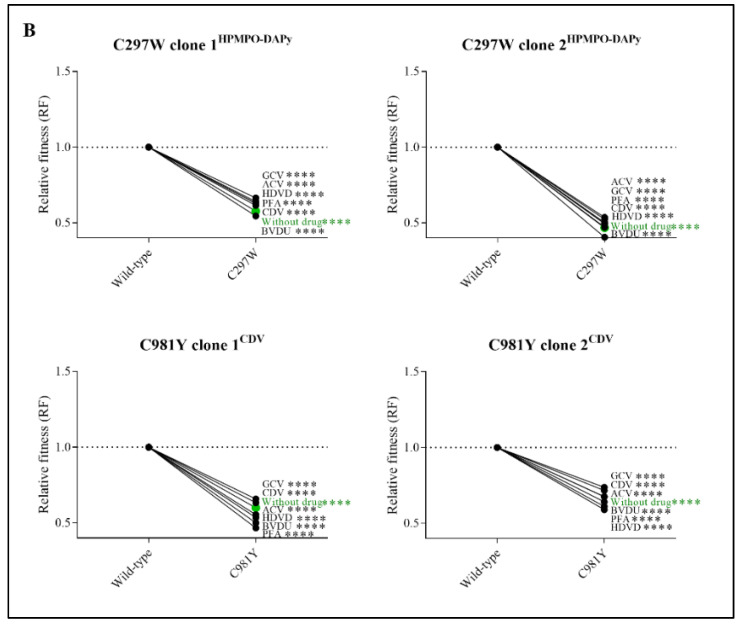
Relative fitness (RF) of MHV-68 wild-type (WT) and drug-resistant clones (**A**) or mutator phenotype viruses (**B**) in growth competition assays in the absence and presence of antiviral drugs. Competition between mutant versus wild-type virus was evaluated without drug (in green) and in the presence of ACV (9 µM), GCV (8 µM), BVDU (1.50 µM), HDVD (0.20 µM), CDV (1.60 µM) or PFA (160 µM). Additionally, the viral mutant Y383S^GCV^ was evaluated with a ratio of 80:20 of Y383S^GCV^/MHV-68 wild-type. Values represent the means of the viral frequencies of two biological replicates, determined by NGS. Statistical significance was calculated using one-way Anova (multiple comparisons to MHV-68 wild-type virus with the Dunnett test. *p* < 0.01 (**), *p* < 0.001 (***), *p* < 0.0001 (****)).

The impact of antiviral treatment on the replication capacity was evaluated by competition of the viruses in the presence of various antiviral drugs (Figure 3A and Table S4). Y383S^GCV^ preserved increased viral fitness under the pressure of ACV, GCV, HDVD, CDV and PFA and was equally as fit as the wild-type virus under the pressure of BVDU. Q827R^HPMP-5azaC^ viral fitness increased under the pressure of GCV, CDV and HPMP-5azaC and diminished in the presence of PFA. The three viral clones selected under PFA (G302W^PFA^, K442T^PFA^ and G302W+K442T^PFA^) increased in viral fitness under the pressure of GCV, HDVD, CDV and PFA. G302W^PFA^ and G302W+K442T^PFA^ populations diminished under the pressure of ACV, while K442T^PFA^ increased under the pressure of ACV.

Due to the increased viral fitness of clone Y383S^GCV^, viral fitness was further investigated using an 80:20 ratio of Y383S^GCV^/MHV-68 wild-type (Figure 3A and Table S4). Altering the ratio of mutant versus wild-type viruses did not affect the viral fitness pattern. Without antiviral pressure, Y383S^GCV^ was more fit compared to the wild-type virus (RF = 1.33, *p* < 0.0001). Additionally, Y383S^GCV^ was more fit under the pressure of antivirals (ACV (RF = 1.49, *p* < 0.0001), GCV (RF = 1.65, *p* < 0.0001), HDVD (RF = 1.44, *p* < 0.0001), CDV (RF = 1.45, *p* < 0.0001) and PFA (RF = 1.52, *p* < 0.0001)), with the exception of BVDU, where both viruses grew equally (RF = 1.07, *p* = 0.17).

### 3.5. Relative Fitness Strongly Correlated with Drug Resistance Levels for Y383S^GCV^, Q827R^HPMP-5azaC^, G302W^PFA^ and G302W+K442T^PFA^, and Moderately for K442T^PFA^

**Figure 4:**
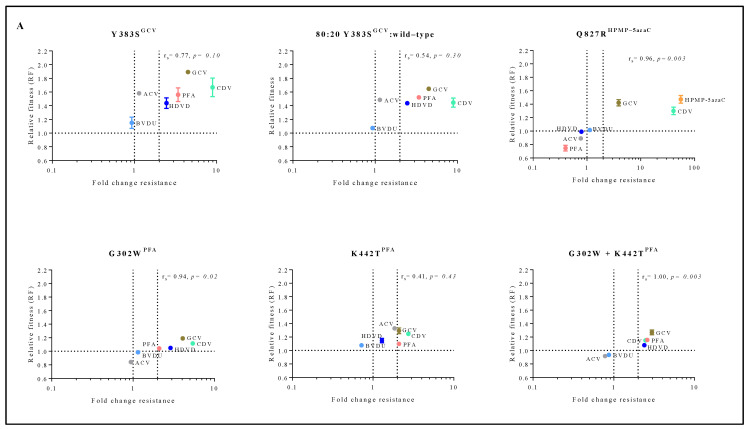

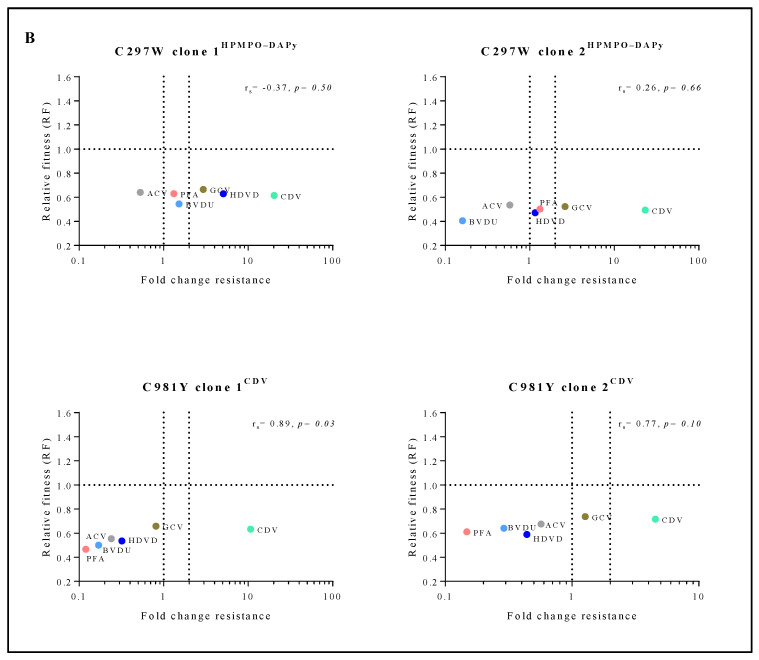
Correlation between relative fitness and drug resistance levels of MHV-68 viral clones bearing amino acid substitutions in the viral DP (**A**) or mutator phenotype viruses (**B**). Spearman’s rank-order correlation test was used to calculate the strength and direction of the relationship between antiviral drug resistance (fold resistance, as shown in Figure 2A,B) and relative fitness (RF, as shown in Figure 3A,B). Spearman’s correlation coefficient (ρ, also signified by r_s_) measures the strength and direction of association between two ranked variables (r_s_ between 0.00 and 0.19 (very weak), 0.20 and 0.39 (weak), 0.40 and 0.59 (moderate), 0.60 and 0.79 (strong) and 0.80 and 1.0 (very strong)).

To understand the connection between viral fitness and drug resistance, changes in viral fitness were analyzed as a function of the fold resistance using Spearman’s rank-order correlation test (Figure 4A). Q827R^HPMP-5azaC^, G302W^PFA^ and G302W+K442T^PFA^ had a very strong positive correlation between relative fitness and the degree of drug resistance (r_s_ = 0.96, *p* < 0.05, r_s_ = 0.94, *p* < 0.05 and r_s_ = 1.00, *p* < 0.05, respectively). Y383S^GCV^ (r_s_ = 0.77, *p* = 0.10) had a strong correlation between relative fitness and drug resistance, and K442T^PFA^ (r_s_ = 0.41, *p* = 0.43) had a moderate correlation.

### 3.6. Mutator Phenotype Viruses Had a Severely Impaired Viral Fitness in the Absence and Presence of Antivirals

Competitive fitness studies for mutator phenotype viruses C297W^HPMPO-DAPy^ and C981Y^CDV^ were performed using two different viral clones (C297W^HPMPO-DAPy^ clones 1 and 2; C981Y^CDV^ clones 1 and 2). Without antiviral pressure, C297W^HPMPO-DAPy^ clones 1 and 2 had a severely impaired replication capacity (RF = 0.58, *p* < 0.0001 and RF= 0.47, *p* < 0.0001, respectively). Likewise, C981Y^CDV^ clones 1 and 2 had a diminished replication capacity (RF = 0.60, *p* < 0.0001 and RF = 0.67, *p* < 0.0001). There was no increase in viral fitness under antiviral pressure (Figure 3B and Table S4).

During dual infection competition experiments, monocultures of both the wild-type virus and resistant virus were included as controls. Monoculture of the C981Y^CDV^ clone 2 in the viral fitness experiments led to the emergence of a second distinct viral population (G302W). Although not present in the original stock, the mutation G302W (±7%) arose in the untreated C981Y^CDV^ clone 2 (93%) viral population. Under the pressure of PFA, the pro-portion of G302W in the population increased to 45% with a corresponding decrease in C981Y to 48%. Under the pressure of HDVD, G302W increased to 21% of the total population (C981Y 74%). Subsequent cloning of the mixed viral population showed that G302W overgrew the mixed viral population, resulting in a pure G302W population with no detectable C981Y nor spontaneous mutations present, indicating an escape mutant. For the three other mutator phenotype viral clones, no escape viruses were detected. 

### 3.7. Mutator Phenotype Virus C981Y^CDV^, but Not C297W^HPMPO-DAPy^, Demonstrated a Correlation between Relative Fitness and Antiviral Drug Resistance Levels

The mutator phenotype viruses C981Y^CDV^ clones 1 and 2 demonstrated a very strong to strong positive correlation between relative fitness and antiviral resistance (r_s_ = 0.89, *p* < 0.05 and r_s_ = 0.77, *p* *=* 0.10, respectively) (Figure 4B). This is in contrast to C297W^HPMPO-DAPy^ clones 1 and 2 that demonstrated a very weak negative (r_s_ = −0.37, *p* *=* 0.50) or positive correlation (r_s_ = 0.26, *p* *=* 0.66), respectively.

### 3.8. Investigation of the Mutation Frequency in the Viral PK, TK and DP

The mutation frequency of three clones of C297W^HPMPO-DAPy^ and three clones of C981Y^CDV^ was investigated by NGS. DNA extracts were prepared at passage 0 and after three rounds of amplification, which allowed evaluating the appearance of new mutations. When comparing the amplified virus to the starting stock, the C297W^HPMPO-DAPy^ clones had 34 newly generated mutations over the span of the viral PK, TK and DP, and the C981Y^CDV^ clones generated 14 new mutations (Table 1).

**Figure 5:**
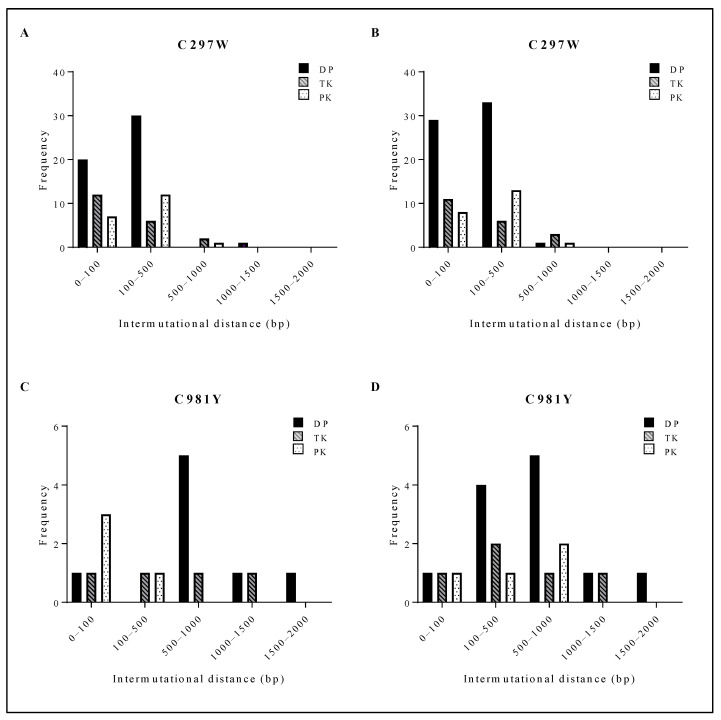
Distribution of the intermutational distance in the viral PK, TK and DP of C297W and C981Y. For both C297W and C981Y, next-generation sequencing of the viral DP, TK and PK was performed on three viral clones at two different points of expansion (i.e., three clones #0 and three clones #3). (**A**) Intermutational distance of 3 viral clones bearing C297W amino acid change without amplification. (**B**) Intermutational distance of 3 viral clones bearing C297W amino acid change after 3 rounds of amplification. (**C**) Intermutational distance of 3 viral clones bearing C981Y amino acid change without amplification. (**D**) Intermutational distance of 3 viral clones bearing C981Y amino acid change after 3 rounds of amplification.

C297W^HPMPO-DAPy^ viral clones had a short intermutational distance, mostly ranging from 0 to 500 bp (Figure 5A,B). C981Y^CDV^ viral clones had a larger intermutational distance from 100 to 1500 bp (Figure 5C,D). Between ungrown (Figure 5A (C297W) and Figure 5C (C981Y)) and amplified viruses (Figure 5B (C297W) and Figure 5D (C981Y)), no shift in intermutational distance was found. The spontaneous mutations arising in C297W^HPMPO-DAPy^ and C981Y^CDV^ viral clones were aligned with HSV-1, HSV-2, VZV and HCMV, and the positions were compared to current information regarding herpesvirus naturally occurring polymorphisms and drug resistance mutations (Tables S5 and S6). Interestingly, various spontaneous mutations were located in conserved regions and were at homologue positions known to confer drug resistance, explaining the variation in drug susceptibility profiles between the two C297W^HPMPO-DAPy^ viral clones (Figure 2B and Table S2).

### 3.9. CRISPR/Cas9 Genome Editing Was a Successful Strategy for the Generation of MHV-68 Virus with a Missense Mutation Resulting in a Mutator Phenotype Virus

**Figure 6:**
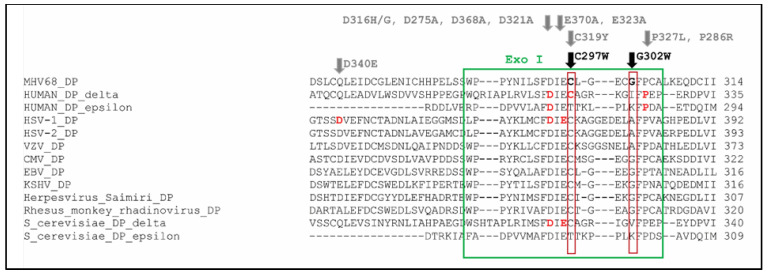
Alignment of MHV-68 DP with human DNA polymerase delta, human DNA polymerase epsilon, HSV-1 DP, HSV-2 DP, VZV DP, HCMV DP, EBV DP, KSHV DP, herpesvirus saimiri DP, rhesus rhadinovirus DP, *S. Cerevisiae* DNA polymerase delta and *S. Cerevisiae* DNA polymerase epsilon. The cysteine at position 297 (marked in bold) is in the Exo I domain (green box) of MHV-68 DP, and it is extremely conserved among B family DNA polymerases. It is part of the FDIEC amino acid sequence, in which amino acid changes are frequently associated with the formation of a mutator phenotype. The amino acid positions associated with a mutator are marked in bold red and encompass the following amino acid changes: human DNA polymerase delta: D316H, D316G, C319Y, P327L; human DNA polymerase epsilon: D275A, P286R; HSV-1 DP: D368A, E370A; *S. Cerevisiae* DNA polymerase delta: D321A, E323A (stated in gray) [51,52,53,54,55,56,57,58,59,60,61,62,63,64,65].

To formally assess the association of the novel identified mutation C297W with a mutator phenotype, i.e., increased frequency of spontaneous mutations, the generation of a recombinant virus is needed to be sure that this mutation actually confers the mutator phenotype. We used a CRISPR/Cas9-based gene editing approach to generate the C297W amino acid change in MHV-68 (Figure S1). The cysteine at position 297 is extremely conserved among α-, β- and γ-herpesviruses (Figure 6). HEK293T cells were transfected with the CRISPR/Cas9 plasmid, and after 24 h, the cells were infected with MHV-68. Multiple concentrations of virus were used as MHV-68 is able to infect HEK293T cells but is not able to generate visible cytopathic effects (CPE). To aid efficient and precise genome editing, the asymmetric template was designed with the PAM sequence as close as possible to the target site.

Our genome editing approach using 25 PFU/well resulted in ~17% efficiency as 3 out of 17 tested clones had the desired mutation (Sanger sequencing). The PK, TK and DP of three C297W^CRISPR^ clones and three wild-type clones (obtained after unsuccessful genome editing) were analyzed by NGS. The three C297W^CRISPR^ clones had spontaneous mutations arising through the viral PK, TK and DP, validating the mutator phenotype (Table S7). Wild-type clones 1 and 2 had no mutations in the viral PK or TK.

Nevertheless, they had a minor fraction with insertions/deletions at position 888 in the viral DP, likely acquired through non-homologous end joining (NHEJ) repair, which is an error-prone DNA repair mechanism, of the Cas9 cut site. Wild-type clone 3 had no mutations in the viral PK, TK, or DP.

## 4. Discussion

In this research, we investigated the resistance of the γ-herpesvirus MHV-68 to several nucleoside (ganciclovir), nucleotide (cidofovir, HPMP-5azaC and HPMPO-DAPy) and pyrophosphate analogues (foscarnet). We evaluated the impact of these mutations on the viral replication capacity by dual infection growth assays and determined the composition of the mixed viral population by NGS. The use of NGS allowed determining the replication capacity of two viral variants under identical conditions with a sensitivity of ≥1%.

The Y383S^GCV^ change is in the exonuclease domain of the viral DP (Exo II) and the viral clone bearing this change had resistance to the purine nucleoside GCV, but not ACV, and cross-resistance to CDV and PFA. Our earlier investigations characterized ACV resistance in MHV-68 and demonstrated a mutation in the activating enzyme (viral PK), but not DP. Similar observations have been made regarding HCMV GCV resistance, where mutations associated with GCV resistance frequently arise in the 3′–5′ exonuclease domain (albeit mutations in the HCMV PK are also reported) [66,67,68]. This could possibly be attributed to the different mechanisms of action of ACV versus GCV. ACV-TP does not possess a hydroxyl group in the 3′ position and is an obligate chain terminator [69], while the presence of a 3′ hydroxyl group in GCV-TP allows for the integration of one additional nucleotide (short-chain terminator) [1].

PFA-resistant clone G302W^PFA^ maps to the exonuclease domain (Exo I), and K442T^PFA^ is adjacent to the δ-C region. The third PFA-resistant viral clone is a double mutant carrying G302W+K442T^PFA^. G302W^PFA^, K442T^PFA^ and G302W+K442T^PFA^ viral clones proved to be resistant to PFA, GCV and CDV, a combination that can be found for mutations located in domain III of the viral DP in HCMV, not in the exonuclease domain or δ-C region [70]. However, for HCMV, few substitutions in the exonuclease region have been implicated in clinical PFA resistance (N495K, D515Y) [71,72] or even observed after PFA exposure in cell culture [73].

C297W^HPMPO-DAPy^ (Exo I) and Q827R^HPMP-5azaC^ (thumb) were resistant to HPMPO-DAPy and HPMP-5azaC, respectively, and cross-resistant to GCV and CDV. This in accordance with reports of HCMV resistance where mutations conferring resistance to ganciclovir and cross-resistance to cidofovir can be found in the exonuclease and thumb domains [74]. C981Y^CDV^, which is also located in the thumb domain, conferred resistance to CDV and other nucleotides, but not to GCV.

Resistance mutations normally arise at the active site of the enzyme, thereby affecting substrate binding and the catalysis rates. Frequently, the affected enzyme is essential to the viral function, which leads to a lower viral fitness [75,76,77]. However, there is scarce information regarding the impact of drug resistance mutations on the viral replication capacity in herpesviruses. Studies on HCMV and HSV-1 demonstrated that drug-resistant herpesviruses have an equal or impaired fitness compared to the wild-type virus, with no reports of increased viral fitness [43,78]. Our earlier work focused on the impact of drug resistance mutations in the MHV-68 viral PK or TK on viral replication capacity and demonstrated that, without antiviral pressure, there was a significant reduction in the viral fitness of a PK:GT-deletion frameshift virus and a decrease, albeit not significant, in the viral fitness of a TK:C-insert frameshift virus [48]. These findings are in line with the current understanding of the functions of the γ-herpesvirus PK and TK, where the viral PK plays an essential role in lytic replication and the viral TK is dispensable [79,80,81,82,83]. The viral DP is an essential enzyme during lytic replication. Nevertheless, in our current research, we showed that in vitro, viruses with a drug resistance mutation in the MHV-68 viral DP had an equal viral fitness compared to the wild-type virus in the absence of antiviral pressure (G302W^PFA^, K442T^PFA^, G302W+K442T^PFA^ and Q827R^HPMP-5azaC^) or increased viral fitness as demonstrated with viral clone Y383S^GCV^ (Figure 3A and Table S4). In the presence of antivirals, drug-resistant viruses had a growth advantage over the wild-type virus, and this growth advantage correlated with antiviral resistance (Figure 4A).

The Y383^GCV^ change maps to the active site of the 3′–5′ exonuclease (Exo II). Investigations by Chen et al. demonstrated that mutations associated with GCV resistance in the HCMV exonuclease domain reduced exonuclease activity, making the polymerase less likely to terminate chain elongation. This allowed further polymerase activity and chain elongation after GCV-TP incorporation and generation of full-length gene products [66]. During DNA synthesis, also correctly incorporated nucleotides are frequently excised [84]. Thus, we can hypothesize that the mutation Y383S^GCV^ affects the exonuclease activity, reducing excision of correct nucleotides and/or elongating the viral DNA after antiviral incorporation, consequently leading to a growth advantage of the drug-resistant viral clone over the wild-type virus.

This study identified C297W^HPMPO-DAPy^ and C981Y^CDV^ as mutator phenotype viruses. Mutator phenotype viruses can arise through different mechanisms as DNA replication fidelity depends on a combination of nucleotide selection, exonucleolytic proofreading and post-replicative mismatch repair (MMR) [52]. Although frequently described in RNA viruses, limited herpesvirus mutator or antimutator phenotypes have been described in the literature (HSV-1 mutators: Y577H+D581A [54], D368A, E370A, D471A, Y538S, U557S, Y557F, D581A [55,69]; CMV mutator: D413A [85,86]). Mutation C297W^HPMPO-DAPy^ is in the 3′–5′ exonuclease domain, a region that harbors many identified mutator positions (Figure S2). Position C297 is extremely conserved among DNA polymerases, i.e., α-, β- and γ-herpesviruses, human DNA polymerase delta, human DNA polymerase epsilon and *S. Cerevisiae* DNA polymerase delta. Moreover, an amino acid change at the exact homologous position (C319) of human DNA polymerase δ was proposed to be the cause of the altered mutation frequency in ultra-hypermutated malignant brain tumors (Figure 6) [64]. Both C297W in MHV-68 DP and C319Y in human DP δ are changes to an aromatic amino acid. The 3′–5′ exonuclease domain has a proofreading capacity, i.e., it catalyzes the removal of mismatched base pairs at the primer terminus, producing deoxyribonucleotide monophosphates [87]. Mutations in the 3′–5′ exonuclease domain can then impair the proofreading capacity and result in an increased mutation frequency, i.e., mutator phenotypes [88]. If the mutations are left unrepaired, they can be propagated in the next round of amplification when the mutated strand acts as a template [51]. In eukaryotes, the distorted sequence is subsequently recognized by DNA mismatch repair mechanisms (MMR) that perform excision and re-synthesis of the nascent strand, a process that is lacking in herpesviruses [63].

C981Y^CDV^, on the other hand, is in the thumb region, for which much fewer mutators and antimutators are known. At this moment, two different mechanisms of mutator generation in the thumb region are described. (i) Mutators in the thumb domain are associated with an altered nucleotide selectivity [89], e.g., in *Sulfolobus solfataricus* DNA polymerase Dpo4, mutation A141D in the thumb domain led to a higher nucleotide incorporation rate, accompanied by an incorrect incorporation of dNTPs, leading to a mutator phenotype virus [90]. (ii) It has been demonstrated that, despite being organized in two different domains, the 3′–5′ exonuclease and thumb domains are structurally and functionally interconnected. More specifically, the putative exonuclease site is in the groove formed between the 3′–5′ exonuclease domain and the tip of the thumb domain [91]. In bacteriophage T4, mutations in the thumb domain (A737V and A777V) were associated with an antimutator phenotype. The mutant strains had an increased hydrolysis of dNTPs per base pair synthesized compared to the wild-type combined with less processivity and less efficient strand displacement synthesis. On the contrary, mutator phenotypes lying in the thumb region hydrolyze less dNTPs per base pair synthesized than the wild-type, indicating the importance of the balance between proofreading and primer extension [52]. It is interesting to note that selection of cidofovir-resistant vaccinia virus also led to a mutator phenotype virus (A684V located in the Pol III catalytic domain) [92].

Monoculture of C981Y^CDV^ clone 3 in the viral fitness experiment led to a second viral population, containing a single G302W mutation in the viral DP. In the untreated dual infection experiment, G302W was present at a low frequency but increased profoundly under the pressure of PFA and HDVD. Interestingly, in this project, we identified C981Y^CDV^ as hypersensitive to PFA and mutation G302W as conferring resistance to PFA (Figure 2 and Table S2). Mutator populations restore the mutation frequency when the mutation rate exceeds 10^−3^ (error-induced extinction). This occurs by acquiring a compensating mutation [54], reversion to the wild-type virus (true revertant) [93], formation of an escape mutant [94] or generating antimutator mutations [51]. In our experiments, G302W was an escape mutant that overgrew C981Y with no detectable C981Y nor remaining spontaneous mutations. The disappearance of C981Y indicated that G302W was not a compensating mutation, and the lack of a detectable wild-type virus excluded a true revertant. The absence of remaining spontaneous mutations and intact viral fitness (Figure 3B and Table S4) indicated that G302W was not an antimutator position.

Our results regarding the severely impaired fitness of MHV-68 mutator phenotype viruses correspond to the current state of the art, i.e., an alteration of the mutation frequency of a virus (mutator or antimutator) results in an impaired replication capacity [95,96]. Additionally, we highlighted that for these viruses, antiviral resistance did not result in increased fitness. Despite that both C297^WHPMPO-DAPy^ and C981Y^CDV^ are mutator viruses, the mutation frequency differs between them, thereby resulting in different strength mutators. C297W showed a higher mutation frequency (Table 1) and a shorter intermutational distance (Figure 5A,B), compared to C981Y (Figure 5C,D).

The fact that different clones of the mutator phenotype viruses have different sensitivity profiles indicates that some of the mutations arising spontaneously contribute to drug resistance. This would have important consequences in the clinic if such mutator phenotypes arise in human herpesviruses.

In viruses, CRISPR/Cas9 genome editing has thus far been used to disrupt specific viral genes [97,98]; however, to our knowledge, this is the first report of a specific amino acid change in a virus generated by CRISPR/Cas9. We validated the mutator phenotype virus associated with amino acid change C297W in the 3′–5′ exonuclease domain by employing a CRISPR/Cas9 approach. Subsequent NGS analysis of the three generated C297W^CRISPR^ clones confirmed the mutator phenotype of the genome-edited clones as spontaneous mutations were identified in the viral PK, TK and DP (Table S7). There were no detectable off-target effects in the rest of the viral genome, demonstrated by NGS of three wild-type viruses obtained after unsuccessful genome editing.

## 5. Conclusions

To summarize, our research contributes to the field of antiviral resistance and allows for a comprehensive investigation of the impact of mutations in the DP on viral fitness. While some mutations confer an intrinsic higher replication capacity such as Y383S^GCV^, others have a growth advantage over the wild-type virus in the presence of antivirals (G302W^PFA^, K442T^PFA^, G302W+K442T^PFA^ and Q827R^HPMP-5azaC^). Our investigations led us to the identification of two mutations associated with a mutator phenotype virus, i.e., C297W^HPMPO-DAPy^, located in the 3′–5′ exonuclease domain, and C981Y^CDV^, located in the thumb domain. Both mutator phenotype viruses had a severely impaired replication capacity, independent from drug resistance patterns. Lastly, we successfully confirmed C297W to be associated with a mutator phenotype virus using CRISPR/Cas9 genome editing.

## Figures and Tables

**Table 1:** Mutation frequency in the viral PK, TK and DP of mutator phenotype viral clones.

Gene	Nucleotide Changes	C297W			C981Y		
		Clone A	Clone B	Clone C	Clone A	Clone B	Clone C
**PK** 1314 bp	A → C					1	
	C → A			1			
	C → T			1			
	G → A						1
	A → T				1	1	
	**Total nucleotide changes**	**0**	**0**	**2**	**1**	**2**	**1**
**TK** 1935 bp	A → G	1			1		
	T → C	1	2				1
	C → A		1	2			
	C → T		1	1			
	G → A		1		1		
	G → T			1			
	A → T					1	
	Frameshift	1					
	**Total nucleotide changes**	**3**	**5**	**4**	**2**	**1**	**1**
**DP** 3084 bp	A → C				1		
	A → G	1		2			
	T → C	2		4	1	1	
	T → G			1	1		
	C → A			2			
	C → T		1				
	G → A			1			
	G → T			1			
	A → T			1	1		
	T → A			1		1	
	Frameshift	1	1	1			
	**Total nucleotide changes**	**4**	**2**	**14**	**4**	**2**	**0**

The mutation frequency of three clones of the mutator viruses C297WHPMPO-DAPy and C981YCDV was investigated by performing NGS on the viral protein kinase (PK), thymidine kinase (TK) and DNA polymerase (DP). DNA extracts were acquired at passage 0 and after 3 rounds of amplification, which allowed evaluating the rise in new mutations. Bold highlights the total nucleotide changes.

## Data Availability

The datasets used and/or analyzed during the current study are available from the corresponding author on reasonable request. All data generated or analyzed during this study are included in this published article (and its Supplementary Information Files).

## Supplementary Materials

The following are available online at https://www.mdpi.com/article/10.3390/v13060985/s1, Figure S1: Steps followed in the generation of C297W DNA polymerase gene-edited virus, Figure S2: Alignment of MHV-68 DP with human DNA polymerase delta, human DNA polymerase epsilon, HSV-1 DP, HSV-2 DP, VZV DP, CMV DP, EBV DP, KSHV DP, herpesvirus saimiri DP, rhesus rhadinovirus DP, *S. Cerevisiae* DNA polymerase delta and *S. Cerevisiae* DNA polymerase epsilon, Table S1: Primers used for PCR amplification and Sanger sequencing of the viral PK, TK and DP, Table S2: The inhibitory effects of a range of antivirals on the replication of MHV-68 wild-type and drug-resistant viruses in Vero cells, Table S3: Spontaneous mutations appearing in the viral PK, TK or DP of different viral clones of C297W and C981Y that led to the hypothesis that amino acid changes C297W and C981Y are associated with mutator phenotype viruses, Table S4: Relative fitness (RF) of MHV-68 wild-type (WT) and drug-resistant clones (4A) or mutator phenotype viruses (4B) in growth competition assays in the absence and presence of antiviral drugs, Table S5: Alignment of C297W spontaneous mutations with HSV-1, HSV-2, VZV and HCMV in the viral PK, TK and DP in the mutation frequency experiment, Table S6: Alignment of C981Y spontaneous mutations with HSV-1, HSV-2, VZV and HCMV in the viral PK, TK and DP in the mutation frequency experiment, Table S7: Alignment of C297W^Crispr^ spontaneous mutations with HSV-1, HSV-2, VZV and HCMV in the viral PK, TK and DP.
