# Supplementary material for: An MHV-68 Mutator Phenotype Mutant Virus, Confirmed by CRISPR/Cas9-Mediated Gene Editing of the Viral DNA Polymerase Gene, Shows Reduced Viral Fitness
Source: Viruses. 2021 May 26;13(6):985. doi: 10.3390/v13060985 (PMC8227558; doi:10.3390/v13060985)
Supplement: Supplementary file 1 [file viruses-13-00985-s001.zip › viruses-1205510-supplementary.pdf]

Supplementary information

Table S1. Primers used for PCR amplification and Sanger sequencing of the viral PK, TK, and DP. The viral PK and TK were PCR amplified using two amplicons (underlined, PK F9-R1162 and F727-R1491; TK F7-R1152 and F770-R1905). The viral DP was amplified using three amplicons (underlined, F12-R1179, F996-R2155 and F1969-R3259). All primers were subsequently used for Sanger sequencing.

	Start nucleotide	Forward primer	Start nucleotide	Reverse primer
**PK**	9	TGTTAACACAGTATCCGCTGCT	636	CCATCAATTAACCCTCTGAAGC
	501	ATGGTGCGTCCAATAAGGTT	1162	CAACAGACAAGCCCCAGACT
	727	GGCAGGAGGACTTTCAGACA	1491	GGGGTGATCTTATAAATGCAGA
**TK**	7	CCTGGAACCTACTGGGTGTG	658	AGTCATCATCAGGCGTTTCC
	494	GGAAATGGCAGGTTCTCCAC	1152	CATGTTCCATCGGAGACCTT
	770	GGAAGAGCATCGGTCAGTTG	1382	TGCAGGTACGTGTGGTTGAT
	1756	TCACAGACCCGGCTATTAAA	1905	ATGCACCAACCACACCATC
**DP**	12	TGTTGTGCTCTCTTTTGTTGAG	929	GGAGAGCTCTGGATGATGACA
	12	ACTTCTATGCCAAGGCTCCA	1179	CGCTGTCCCTCATGAAAATTA
	996	GTGAATGTGGGTTTCCCTGT	1664	CATCAGTCAGCACCCGTCT
	1476	GCCTGAACATGGAAAAGGAA	2155	CAGAGGCCACTCCTGTGAAG
	1969	TAGCGGCACTGTTCACTTTG	2649	CAGCTTCCCCCAGTTACTCA
	2538	AGGGTGTGGATCTTGTCAGG	3259	CCAGAGTGGTGTATCCATTCC

Table S2. The inhibitory effects of a range of antivirals on the replication of MHV-68 wild type and drug-resistant viruses in Vero cells. MHV-68 wild-type and drug-resistant viral clones were characterized phenotypically by cytopathic effect (CPE) reduction assays. For each compound, the 50% effective concentration (EC_{50}), i.e. compound concentration required to reduce virus-induced CPE by 50%, was calculated. For each viral mutant, minimum 3 independent phenotypic assays were performed, and the mean EC_{50} values were calculated.

2A

**MHV-68 genotype**	**EC_{50} (µM)**									
	**Purine nucleoside analogues**		**Pyrimidine nucleoside analogues**				**Acyclic nucleoside phosphonates**			**Pyrophosphate analogue**
	**ACV**	**GCV**	**BVDU**	**HDVD**	**KAH-39-139**	**KAY-2-41**	**CDV**	**HPMP-5azaC**	**HPMPO-DAPy**	**PFA**
MHV-68 wild-type	4.54 ± 1.66	9.58 ± 1.34	1.93 ± 0.64	0.65 ± 0.21	1.91 ± 0.64	53.02 ± 11.81	2.62 ± 0.55	1.74 ± 0.51	0.36 ± 0.07	301.67 ± 34.58
Y383S^{GCV}	5.16 ± 1.57	42.91 ± 9.47	1.79 ± 0.78	1.59 ± 0.49	≤ 0.18 ± 0.05	> 183.6.1 ±0.00	23.39 ± 8.85	9.47 ± 2.98	7.73 ± 2.57	1027.16 ± 408.39
Q827R$^{HPMP\text{-}5azaC}$	3.50 ± 1.10	37.20 ±8.68	2.17 ± 1.07	≤0.51 ± 0.26	2.62 ± 1.18	>183.61 ± 0.00	≥106.14 ±47.21	96.93 ± 25.20	8.44 ± 2.45	120.34 ± 27.01
G302W^{PFA}	4.25 ± 0.24	39.08 ± 1.90	2.22 ± 0.84	1.86± 0.52	10.09 ± 1.91	83.92 ± 25.70	14.23 ± 3.22	3.82 ± 2.01	3.13 ± 2.12	633.85 ± 44.32
K442T^{PFA}	8.39 ± 0.72	20.07 ± 3.90	1.39 ± 0.31	≤0.83 ± 0.26	2.27 ± 1.14	28.34 ± 9.44	7.19 ± 1.57	3.76 ± 2.21	0.74 ± 0.39	632.94 ± 44.51
G302W+K442T^{PFA}	3.54 ± 1.04	28.40 ± 1.08	1.67 ± 1.01	1.54 ± 1.14	1.19 ± 0.25	≥ 147.26 ± 34.41	6.47 ± 0.34	3.48 ± 0.69	2.41 ± 0.75	783.78 ± 124.05

2B

**MHV-68 genotype**	**EC_{50} (µM)**									
	**Purine nucleoside analogues**		**Pyrimidine nucleoside analogues**				**Acyclic nucleoside phosphonates**			**Pyrophosphate analogue**
	**ACV**	**GCV**	**BVDU**	**HDVD**	**KAH-39-139**	**KAY-2-41**	**CDV**	**HPMP-5azaC**	**HPMPO-DAPy**	**PFA**
C297W clone 1$^{HPMPO\text{-}DAPy}$	2.43 ± 0.79	28.14 ±10.25	2.94 ± 1.74	3.28 ± 2.56	≤0.20 ± 0.09	≥123.95±55.35	53.25 ± 21.54	30.44 ± 15.49	20.40 ± 12.53	>396.83 ± 0.00
C297W clone 2$^{HPMPO\text{-}DAPy}$	2.65 ± 1.70	25.13 ±11.65	0.32 ± 0.07	0.75 ± 0.49	<0.15 ± 0.00	56.38 ± 45.10	61.00 ± 26.73	17.56 ± 5.93	24.04 ± 11.74	>396.83 ± 0.00
C981Y clone 1^{CDV}	1.09 ± 0.17	7.79 ± 2.32	0.33 ± 0.14	<0.21 ± 0.00	<0.15 ± 0.00	47.22 ± 33.45	28.08 ± 12.61	16.15 ± 4.50	3.12 ± 1.09	35.68 ± 17.09
C981Y clone 2^{CDV}	2.57 ± 0.30	12.14 ± 2.08	0.57 ± 0.21	≤0.29 ± 0.13	≤0.21 ± 0.10	36.07 ± 10.95	11.91 ± 3.58	4.59 ± 0.47	2.44 ± 0.47	44.53 ± 8.92

Table S3. Spontaneous mutations appearing in the viral PK, TK, or DP of different viral clones of C297W and C981Y that led to the hypothesis that amino acid changes C297W and C981Y are associated with a mutator phenotype virus. Viral clones C297W and C981Y were isolated through limited dilution. **(A)** Sanger sequencing of 6 clones of C297W demonstrated various mutations in the viral PK, TK, or DP. **(B)** The mutator virus phenotype of C981Y was determined using NGS and demonstrated various mutations in the viral DP of 2 viral clones.

A

C297W	MHV-68 Δ nucleotide	MHV-68 Δ amino acid
PK	A133G	M45V
	G432A	M144I
	G643A	D215N
	G1018A	A340T
	A1024G	M342V
TK	G632T	R211M
	T636G	S212A
	G1135A	V379I
	A1401G	K467 (silent)
	G1519T	V507L
	G1546A	E516K
	G1551A	L517(silent)
	C1560G	I520 (silent)
	C1568A	P523H
	C1900A	L634I
	T1920C	G640(silent)
DP	C544A	H182N
	A565T	I189F
	C568T	P190S
	C579A	T193(silent)
	C849A	S283(silent)
	G935A	C312Y
	T891G	C297W
	C1010T	T337I
	T1088C	M363T
	C1418A	P473Q
	G1968A	Q656 (silent)
	A1993G	T665A
	C2350A	L784I
	T2450C	L817P
	G2582T	R861M
	A2970C	T990 (silent)

B

C981Y	MHV-68 Δ nucleotide	MHV-68 Δ amino acid
DP	T159C	L53 (silent)
	T291C	H97 (silent)
	G904T	G302W (PFA-resistant)
	C1014T	C338 (silent)
	A1336G	S446G
	A1522G	I508V
	A2110G	T704A
	A2410G	T804A
	T2836C	S946P
	G2942A	C981Y

Table S4: Relative fitness (RF) of MHV-68 wild-type (WT) and drug-resistant clones (A) or mutator phenotype viruses (B) in growth competition assays in the absence and presence of antiviral drugs. Vero cells were infected with 750 PFU/well of MHV-68 wild-type, a drug-resistant viral clone or a 50:50 ratio of both viruses. After 2 h incubation, residual virus was removed and fresh medium with or without antivirals was added to the cell cultures. The following antivirals were used: 9 µM ACV, 8 µM GCV, 1.50 µM BVDU, 0.20 µM HDVD, 1.60 µM CDV or 160 µM PFA. The day of infection (day 0) and after 5 days of growth, an aliquot of each viral inoculum was used for DNA extraction to determine viral variants frequency by NGS and using this data the relative fitness was calculated. An RF value of 1 represents equivalent levels of viral fitness between the competitors; a value of <1 signifies less growth of the mutant viral variant than that of the wild-type virus; a value >1 indicates a more efficient grown of the mutant viral variant than that of the wild-type virus. The data shown represent the mean RF-values from two biological replicates. [b]*P* values were determined using one-way Anova [multiple comparisons compared to MHV-68 wild-type with Dunnett test (P ≤ 0.01)].

**A**				
**Competition condition and genotype of competing viruses**	**Antiviral**	**Relative fitness (RF) value**[a]	***P* value**[b]	**Fitness interpretation (mutant *vs* WT)**
WT *vs* WT		1.00 (±0.0000)		=
$Y383S^{GCV}$ *vs* WT	None	1.33 ± 0.013	0.0090	>
	ACV	1.58 ± 0.0003	0.0002	>
	GCV	1.89 ± 0.0014	<0.0001	>
	BVDU	1.15 ± 0.083	0.2647	=
	HDVD	1.44 ± 0.078	0.0016	>
	CDV	1.67 ± 0.14	0.0001	>
	PFA	1.56 ± 0.099	0.0003	>
80:20 $Y383S^{GCV}$ *vs* WT	None	1.33 ± 0.041	<0.0001	>
	ACV	1.49 ± 0.0011	<0.0001	>
	GCV	1.65 ± 0.028	<0.0001	>
	BVDU	1.07 ± 0.013	0.1680	=
	HDVD	1.44 ±0.0052	<0.0001	>
	CDV	1.45 ± 0.068	<0.0001	>
	PFA	1.52 ±0.0042	<0.0001	>
$Q827R^{HPMP\text{-}5azaC}$ *vs* WT	None	1.08 ± 0.017	0.2155	=
	ACV	0.89 ± 0.011	0.063	=
	GCV	1.42 ± 0.047	<0.0001	>
	BVDU	1.02 ± 0.0002	0.9976	=
	HDVD	0.99 ± 0.026	0.9996	=
	CDV	1.30 ± 0.055	0.0001	>
	HPMP-5azaC	1.47 ±0.055	<0.0001	>
	PFA	0.75 ± 0.041	0.0003	<
$G302W^{PFA}$ *vs* WT	None	0.98 ± 0.013	0.2405	=
	ACV	0.84 ± 0.006	<0.0001	<
	GCV	1.19 ± 0.0012	<0.0001	>
	BVDU	0.98 ± 0.0069	0.3628	=
	HDVD	1.05 ± 0.014	0.0024	>
	CDV	1.12 ± 0.0060	<0.0001	>

	PFA	1.04 ± 0.0095	0.0062	>
K442T^{PFA} *vs* WT	None	1.02 ± 0.012	0.8177	=
	ACV	1.33 ± 0.011	<0.0001	>
	GCV	1.29 ± 0.012	<0.0001	>
	BVDU	1.08 ± 0.012	0.0311	=
	HDVD	1.15 ± 0.033	0.0006	>
	CDV	1.25 ± 0.0008	<0.0001	>
	PFA	1.10 ± 0.0152	0.0081	>
G302W+K442T^{PFA} *vs* WT	None	0.97 ± 0.020	0.4780	=
	ACV	0.92 ± 0.0099	0.004	<
	GCV	1.27 ± 0.035	<0.0001	>
	BVDU	0.93 ± 0.012	0.0156	=
	HDVD	1.08 ± 0.0060	0.0063	>
	CDV	1.15 ± 0.0086	<0.0001	>
	PFA	1.16 ± 0.0097	<0.0001	>

B

**Competition condition and genotype of competing viruses**	**Antiviral**	**Relative fitness (RF) value[a]**	***P* value[b]**	**Fitness interpretation (mutant *vs* WT)**
WT *vs* WT		1.00 (±0.0000)		=
C297W clone 1$^{HPMPO-DAPY}$ *vs* WT	None	0.58± 0.0030	<0.0001	<
	ACV	0.64 ± 0.0062	<0.0001	<
	GCV	0.66 ±0.013	<0.0001	<
	BVDU	0.54 ± 0.0040	<0.0001	<
	HDVD	0.63 ± 0.0029	<0.0001	<
	CDV	0.61 ± 0.016	<0.0001	<
	PFA	0.63 ± 0.0040	<0.0001	<
C297W clone 2$^{HPMPO-DAPY}$ *vs* WT	None	0.47 ± 0.0053	<0.0001	<
	ACV	0.54 ± 0.011	<0.0001	<
	GCV	0.52 ± 0.0065	<0.0001	<
	BVDU	0.40 ± 0.0078	<0.0001	<
	HDVD	0.47 ± 0.0022	<0.0001	<
	CDV	0.49 ± 0.0022	<0.0001	<
	PFA	0.50 ± 0.020	<0.0001	<
C981Y clone 1^{CDV} *vs* WT	None	0.60 ± 0.012	<0.0001	<
	ACV	0.55 ± 0.0019	<0.0001	<
	GCV	0.66 ± 0.014	<0.0001	<
	BVDU	0.50 ± 0.013	<0.0001	<
	HDVD	0.54 ± 0.011	<0.0001	<
	CDV	0.63 ± 0.011	<0.0001	<
	PFA	0.47 ± 0.022	<0.0001	<
C981Y clone 2^{CDV} *vs* WT	None	0.67 ± 0.0086	<0.0001	<
	ACV	0.68 ± 0.019	<0.0001	<
	GCV	0.74 ± 0.014	<0.0001	<
	BVDU	0.64 ± 0.0051	<0.0001	<
	HDVD	0.59 ± 0.013	<0.0001	<
	CDV	0.72 ± 0.0013	<0.0001	<
	PFA	0.61 ± 0.0095	<0.0001	<

Table S5. Alignment of C297W spontaneous mutations with HSV-1, HSV-2, VZV and CMV in the viral PK, TK, and DP in the mutation frequency experiment. Known polymorphisms are marked in yellow polymorphisms, known mutations are marked in red and conserved positions are underlined in italic.

	MHV-68 Δ nucleotide	MHV-68 Δ amino acid	Corresponding position in			
			HSV-1	HSV-2	VZV	CMV
PK	A133G	M45V	-	-	-	S306
	A332C	*K111T*	-	-	-	*K359*
	T344C	L115P	-	-	-	-
	C459A	I153 (silent)	-	-	-	T409
	A del 468	P156fs	-	-	-	R412
	A630C	A210 (silent)	-	-	-	V466
	T ins 769	F257fs	-	-	-	V498
	C776T	A259V	-	-	-	Q500
	G1018A	A340T	-	-	-	R593
	A1104G	G368 (silent)	-	-	-	G623
	G1209A	R403 (silent)	-	-	-	S657
	G1246A	V416M	-	-	-	E670
	C1281A	C427*	-	-	-	S676
TK	G157T	E53*	-	-	-	-
	C389A	S130Y	-	-	-	-
	C414A	I138 (silent)	-	-	-	-
	C489A	P163 (silent)	-	-	-	-
	C646T	Q216*	-	-	-	-
	C1162T	H388Y	S113	S114	S78	-
	T1206C	*P402 (silent)*	*P131*	*P132*	*P96*	-
	A1354G	R452G	T183	T184	S150	-
	C1396T	Q466*	T197	T198	E164	-
	A1436G	K479R	D211	A212	P178	-
	A1447T	T483S	D215	D216	S182	-
	T ins 1532	*I511 FS*	*L242*	*L243*	*L209*	-
	G1546A	E516K	G260	G261	N226	-
	C1568A	P523H	V267	A268	D233	-
	A1676G	*D559G*	*D286*	*D287*	*D255*	-
	G1699A	E567K	-	-	-	-
	C1730T	*S577L*	*A299*	*A300*	*G268*	-
	T1797C	*D599 (silent)*	*D328*	*D329*	*S297*	-
DP	C27T	R9 (silent)	A50	A51	T13	S58
	C97T	*L33 (silent)*	*I77*	*I78*	*L46*	*I44*
	T159C	L53 (silent)	R101	R102	R70	R64
	G244A	A82T	V133	A134	I102	-
	C248T	S83L	D134	D135	D103	-
	C380A	*T127N*	*T180*	*T181*	*T145*	*N132*
	G382A	*V128I*	*V181*	*V182*	*V146*	*V133*
	T383C	*V128A*	*V181*	*V182*	*V146*	*V133*
	G414A	*G138 (silent)*	*G191*	*G192*	*G156*	*G143*
	A ins 443	Q148fs	T201	T202	I166	Q153
	T479C	L160P	D213	D214	D178	D165
	G del 530	R177 fs	-	-	-	R182
	A565T	I189F	T256	A257	T237	T195
	C568T	P190S	D257	D258	D238	S196
	T657C	*L219 (silent)*	*F286*	*F287*	*F267*	*L226*
	G701A	C234Y	T300	T301	T281	L241
	A748G	T250A	R317	R318	Q298	S257
	T891G	*C297W*	*C371*	*C372*	*C352*	*C304*
	G930T	*Q310H*	*E388*	*E389*	*E369*	*D318*
	C1010T	*T337I*	*S416*	*S417*	*S397*	*T369*
	T1208A	V403D	N494	N495	N475	P436
	A1272C	Q424H	R512	R513	R493	A473
	A1293C	*G431 (silent)*	*G519*	*G520*	*G500*	*G480*
	C1418A	*P473Q*	*P561*	*P562*	*P542*	*P522*
	A1698G	*I566M*	*L702*	*L707*	*F667*	*F702*
	T1733C	L578P	V714	V719	V679	A714
	T ins 1857	T619fs	E754	E759	T718	S752
	A1930G	T644A	R779	R784	K743	N778

G1968A	Q656 (silent)	R791	R796	R755	C790
A1997C	K666T	A800	A805	A764	R800
T2090C	*L697P*	*L831*	*L836*	*L795*	*L831*
T2230C	*Y744H*	*Y884*	*Y889*	*Y849*	*Y905*
C2350A	*L784I*	*L924*	*L929*	*L889*	*L948*
C2 del 350	L784 fs	L924	L929	L889	L948
A2494G	N832D	A972	A977	937K	D997
T2700C	P900 (silent)	H1040	H1045	P1005	D1065
A2761G	*R921G*	*R1061*	*R1066*	*R1026*	*R1086*
C2777A	*P926H*	*P1066*	*P1071*	*P1031*	*P1091*
T2795C	*I932T*	*I1072*	*I1077*	*I1037*	*V1097*
G2882T	R961I	A1155	P1160	S1096	P1183
A2970C	T990 (silent)	T1184	T1189	R1125	R1212
C2997T	N999 (silent)	-	-	-	-
C3031T	*L1011F*	*V1202*	*V1207*	*M1243*	*F1231*

Table S6. Alignment of C981Y spontaneous mutations with HSV-1, HSV-2, VZV and CMV in the viral PK, TK, and DP in the mutation frequency experiment. Known polymorphisms are marked in yellow polymorphisms, known mutations are marked in red and conserved positions are underlined in italic.

	MHV-68 Δ nucleotide	MHV-68 Δ amino acid	Corresponding position in HSV-1	HSV-2	VZV	CMV
PK	A123T	K41N	-	-	-	F293
	C197A	*S66**	-	-	-	*D319*
	G626A	*G209E*	-	-	-	*D465*
	T707C	I236T	-	-	-	Y487
	G1002A	*R334 (silent)*	-	-	-	*H587*
TK	G72A	A24 (silent)	-	-	-	-
	T267C	S89 (silent)	-	-	-	-
	A794G	K265R	Q8	Q8	-	-
	A1401T	K467N	L198	A199	P165	-
	G1590A	M530I	-	-	-	-
DP	G207A	Q69 (silent)	V117	V118	F86	L77
	A565T	I189F	T256	A257	T237	T195
	T726A	N242	G309	G310	G290	R249
	C828A	*I276 (silent)*	*L350*	*L351*	*L331*	*L283*
	C1014T	*C338 (silent)*	*C417*	*C418*	*C398*	*C370*
	A1336G	S446G	K534	K535	K515	N495
	A1522G	I508V	L596	L597	L577	Y557
	T1556C	*I519T*	*I607*	*I608*	*I588*	*I568*
	C1691A	T564K	K700	R705	R665	T700
	A2410G	T804A	V944	V949	V909	K968
	A del 2410	T804 fs	V944	V949	V909	K968
	T2448C	*D816 (silent)*	*D956*	*D961*	*D921*	*D981*
	T2778G	*P926 (silent)*	*P1066*	*P1071*	*P1031*	*P1091*
	G2942A	C981Y	A1175	A1180	A1116	P1203

Table S7. Alignment of C297W^{Crispr} spontaneous mutations with HSV-1, HSV-2, VZV and CMV in the viral PK, TK, and DP. Known polymorphisms are marked in yellow polymorphisms, known mutations are marked in red and conserved positions are underlined in italic.

	MHV-68 Δ nucleotide	MHV-68 Δ amino acid	Corresponding position in HSV-1	HSV-2	VZV	CMV
PK	C62A	S21Y	-	-	-	C274
	G408A	A136 (silent)	-	-	-	V392
	A706G	I236M	-	-	-	Y488
	T ins 769	F257fs	-	-	-	V498
	G822T	K274N	-	-	-	R524
	T843C	A281 (silent)	-	-	-	L531
	T904C	C302R	-	-	-	A555
TK	A120G	G40 (silent)	-	-	-	-
	G476T	R159L	-	-	-	-
	C869T	T290M	H22	P22	-	-
	C1053T	H351 (silent)	D76	D77	N41	-
	A1741G	T581A	D303	D304	N272	-
DP	C214T	Q72*	G120	E121	D80	S89
	G382A	*V128I*	*V181*	*V182*	*V133*	*V146*
	T383C	*V128A*	*V181*	*V182*	*V133*	*V146*
	T1766C	*I589*	*I725*	*I730*	*I725*	*I690*
	G2165C	S722T	W856	W861	F856	W820
	G2379A	*L793 (silent)*	*L933*	*L938*	*L957*	*L898*
	G2821T	E941*	R1081	R1086	V1106	D1046

Supplementary Figure S1. Steps followed in the generation of C297W DNA polymerase gene edited virus

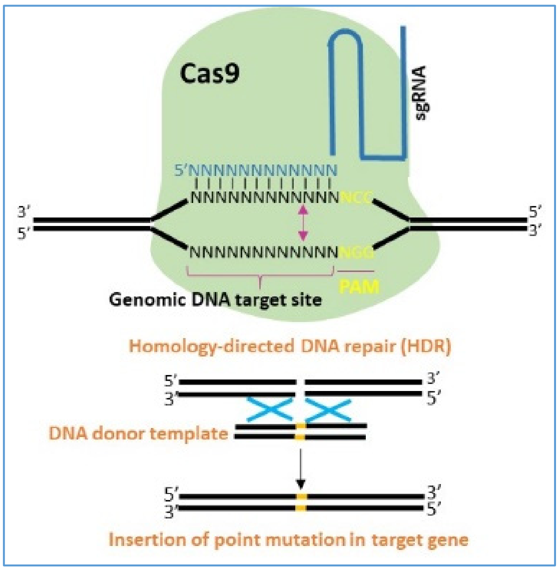

(I). Schema of CRISPR/Cas9-mediated genome editing. The 20nt single-guide RNA (sgRNA) guides the Cas9 nuclease to the complementary genomic target locus, which must be located next to the protospacer adjacent motif (PAM). The PAM is a short DNA sequence (usually 2-6 base pairs in length) that is recognized by Cas9 nuclease and is generally found 3-4 nucleotides downstream from the cut site. Cas9 unwinds the DNA duplex and cleaves both strands to form a **double-strand break (DSB)**. DSBs can be repaired by non-homologous end-joining (NHEJ) or **homology-directed repair (HDR)** using a donor DNA template [i.e. single-stranded oligo DNA nucleotides (ssODNs)]. While NHEJ is used for disruption of a target gene by generating premature stop codons or frameshifts, HDR is used to introduce single-base substitutions.

(II). Donor asymmetric templates designed and used for generation of the C297W MHV-68 recombinant viruses. The asymmetric templates carry the nucleotide change corresponding to MHV-68 T891G mutation. For the generation of MHV-68 C297W recombinant viruses, we first designed the donor asymmetric template depicted in **(A).** Three out of 44 screened MHV-68 viral clones showed the silent change in the PAM sequence, but not the desired edited single-base substitution, indicating that the Cas9 nuclease did cut the DNA duplex and HDR occurred but recombination events occurred without including the target base change. Then, a new asymmetric template was designed with the PAM sequence within a shorter distance from the base-pair substitution relative to Cas9 cut site **(B),** which resulted in the generation of 3 viral clones bearing C297W substitution.

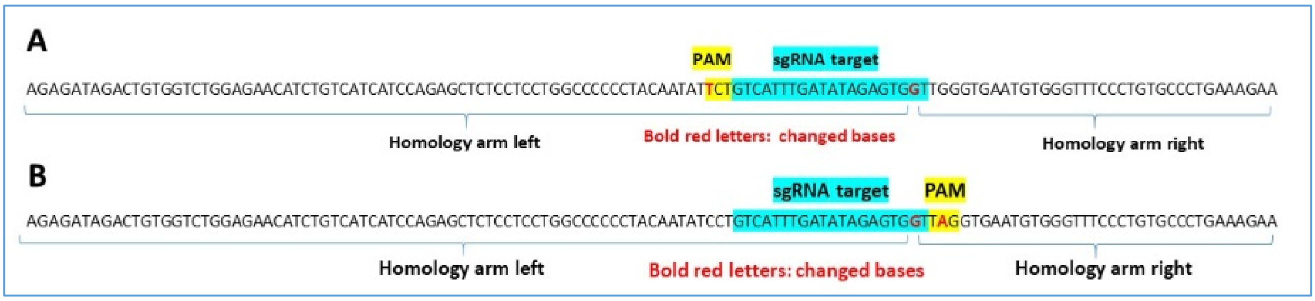

(III) Steps for the construction of DNA pol gene edited MHV-68. A) Plasmid construction - The GeneArt® CRISPR Nuclease Vector with OFP (orange fluorescence protein) Reporter Kit (Thermo Fisher Scientific), encoding the Cas9 endonuclease and a target-specific user-defined guide RNA (gRNA), was used to edit the genomic sequence of MHV-68 DNA pol with the desired changes. Briefly, the plasmid encoding the target RNA of interest was amplified in TOP10 E. coli cells and purified using the PureLink HiPure Plasmid Midiprep kit (Invitrogen). A template carrying the sought mutation was designed (Eurofins Genomics). Additionally, the PAM site was altered with a silent mutation in this template to avoid further cleavage by the CRISPR/Cas9 system. **B) Transfection GeneArt vector and template followed by viral infection -** The nuclease vector and its associated template were transfected into HEK293T cells) using Lipofectamine 3000 (Thermo Fisher Scientific).

Transfection efficiency was assessed (detection of OFP by immunofluorescence and flow cytometry). After 24h, the cells were infected with MHV-68 for 2h, followed by removal of the viral inoculum and incubation with fresh medium. Cell culture supernatants were harvested and viral clones were isolated by plaque assay in Vero cells. **C) Screening of viral clones and selection of clones with the desired mutations -** Individual viral clones were collected and screened for the desired mutation by Sanger sequencing. Viral stocks were prepared in Vero cells for characterization of the recombinant viruses.

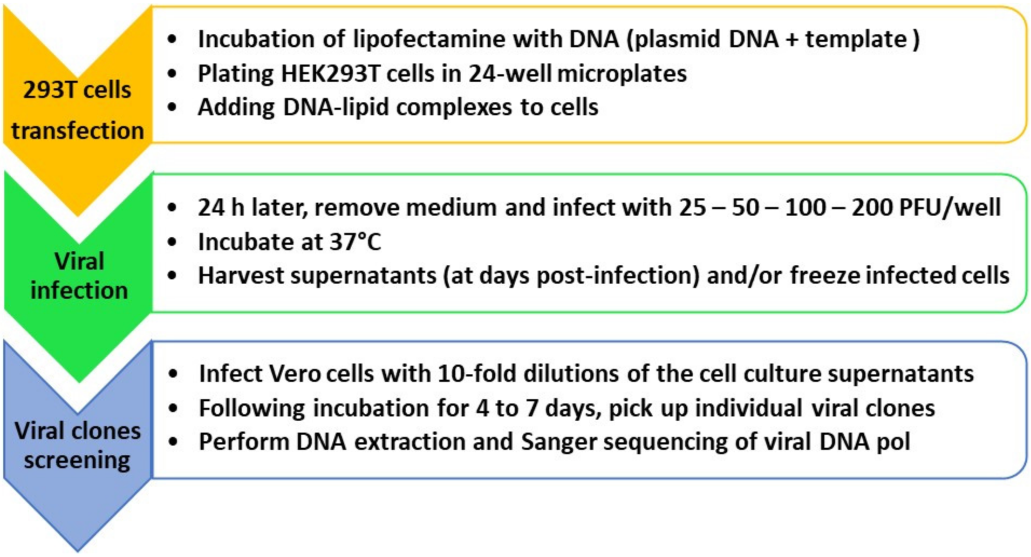

Supplementary Figure S2. Alignment of MHV-68 DP with human DNA polymerase delta, human DNA polymerase epsilon, HSV-1 DP, HSV-2 DP, VZV DP, CMV DP, EBV DP, KSHV DP, herpesvirus saimiri DP, rhesus rhadinovirus DP, *S. Cerevisiae* DNA polymerase delta and *S. Cerevisiae* DNA polymerase epsilon. The different domains correspond to Exo I; II and II (green boxes), Pol IV, II, VI, III and IV (gold boxes) and δ-C-region (purple box) (1-4). The mutations selected under pressure of ganciclovir, cidofovir, HPMP-5azaC and HPMPO-DAPy are marked stated above the alignment in black and the positions are marked in the alignment in bold black. Known mutator and antimutator positions are stated above the alignment in grey and blue respectively (5-17). The positions are highlighted in bold red in the alignments.

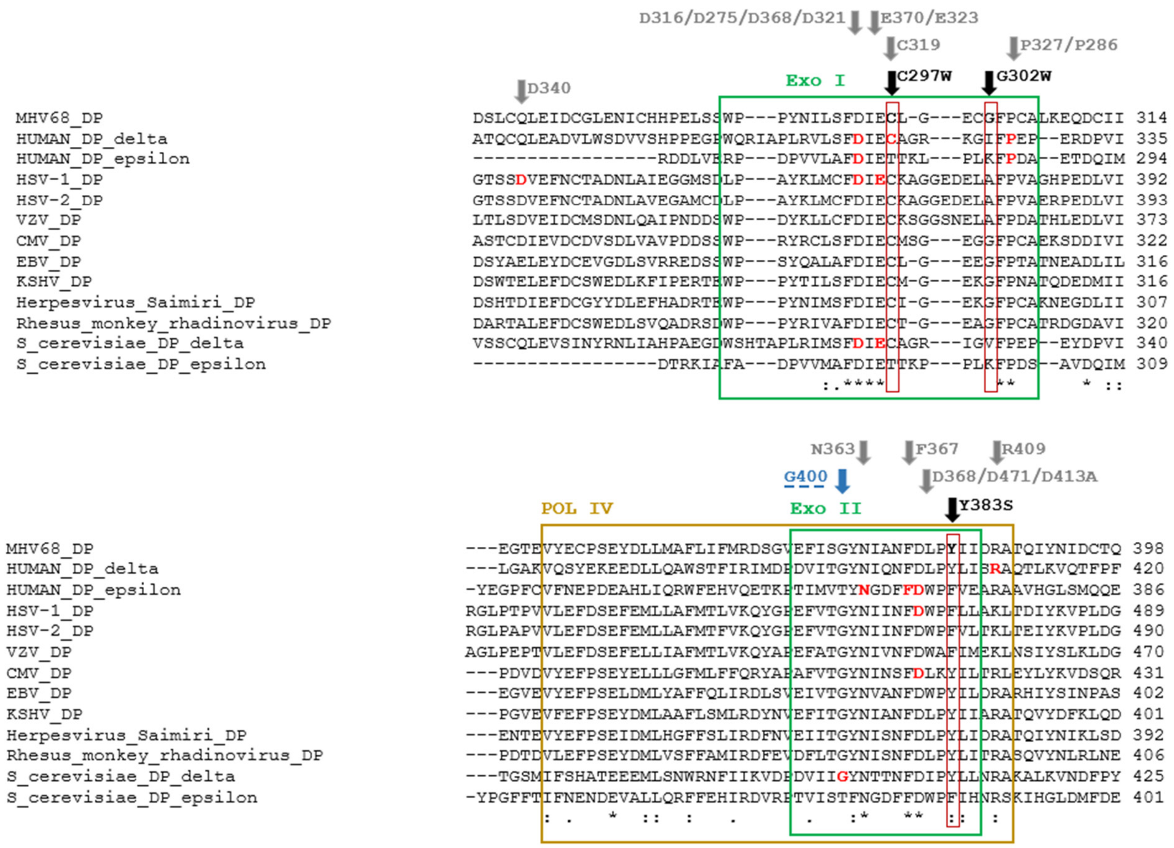

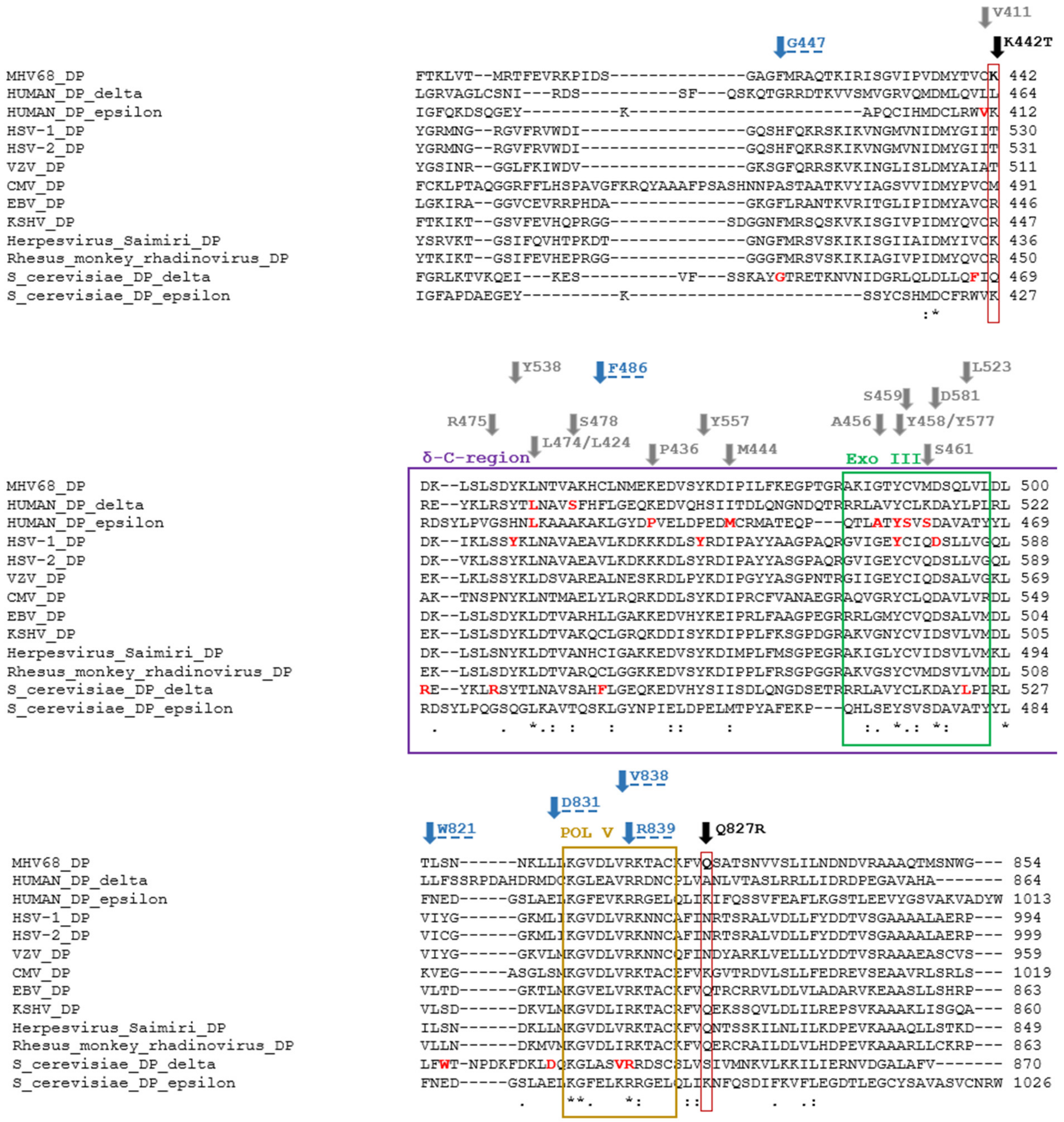
V411
G447
K442T
MHV68_DP FTKLVT--MRTFEVRKPIDS--------------GAGFMRAQTKIRISGVIPVDMYTVCK 442
HUMAN_DP_delta LGRVAGLCSNI---RDS----------SF---QSKQTGRRDTKVVSMVGRVQMDMLQVLL 464
HUMAN_DP_epsilon IGFQKDSQGEY----------K------------------------APQCIHMDCLRWVK 412
HSV-1_DP YGRMNG--RGVFRVWDI-----------------GQSHFQKRSKIKVNGMVNIDMYGIIT 530
HSV-2_DP YGRMNG--RGVFRVWDI-----------------GQSHFQKRSKIKVNGMVNIDMYGIIT 531
VZV_DP YGSINR--GGLFKIWDV-----------------GKSGFQRRSKVKINGLISLDMYAIAT 511
CMV_DP FCKLPTAQGGRFFLHSPAVGFKRQYAAAFPSASHNNPASTAATKVYIAGSVVIDMYPVCM 491
EBV_DP LGKIRA--GGVCEVRRPHDA--------------GKGFLRANTKVRITGLIPIDMYAVCR 446
KSHV_DP FTKIKT--GSVFEVHQPRGG------------SDGGNFMRSQSKVKISGIVPIDMYQVCR 447
Herpesvirus_Saimiri_DP YSRVKT--GSIFQVHTPKDT--------------GNGFMRSVSKIKISGIIAIDMYIVCK 436
Rhesus_monkey_rhadinovirus_DP YTKIKT--GSIFEVHEPRGG--------------GGGFMRSVSKIKIAGIVPIDMYQVCR 450
S_cerevisiae_DP_delta FGRLKTVKQEI---KES----------VF---SSKAYGTRETKNVNIDGRLQLDLLQFIQ 469
S_cerevisiae_DP_epsilon IGFAPDAEGEY----------K------------------------SSYCSHMDCFRWVK 427
:*
Y538
F486
L523
S459
D581
R475
S478
Y557
A456
Y458/Y577
L474/L424
P436
M444
S461
δ-C-region
Exo III
MHV68_DP DK--LSLSDYKLNTVAKHCLNMEKEDVSYKDIPILFKEGPTGRAKIGTYCVMDSQLVIDL 500
HUMAN_DP_delta RE--YKLRSYTLNAVSFHFLGEQKEDVQHSIITDLQNGNDQTRRRLAVYCLKDAYLPLRL 522
HUMAN_DP_epsilon RDSYLPVGSHNLKAAAKAKLGYDPVELDPEDMCRMATEQP---QTLATYSVSDAVATYYL 469
HSV-1_DP DK--IKLSSYKLNAVAEAVLKDKKKDLSYRDIPAYYAAGPAQRGVIGEYCIQDSLLVGQL 588
HSV-2_DP DK--VKLSSYKLNAVAEAVLKDKKKDLSYRDIPAYYASGPAQRGVIGEYCVQDSLLVGQL 589
VZV_DP EK--LKLSSYKLDSVAREALNESKRDLPYKDIPGYYASGPNTRGIIGEYCIQDSALVGKL 569
CMV_DP AK--TNSPNYKLNTMAELYLRQRKDDLSYKDIPRCFVANAEGRAQVGRYCLQDAVLVRDL 549
EBV_DP DK--LSLSDYKLDTVARHLLGAKKEDVHYKEIPRLFAAGPEGRRRLGMYCVQDSALVMDL 504
KSHV_DP EK--LSLSDYKLDTVAKQCLGRQKDDISYKDIPPLFKSGPDGRAKVGNYCVIDSVLVMDL 505
Herpesvirus_Saimiri_DP DK--LSLSNYKLDTVANHCIGAKKEDVSYKDIMPLFMSGPEGRAKIGLYCVIDSVLVMKL 494
Rhesus_monkey_rhadinovirus_DP EK--LSLSDYKLDTVARQCLGGKKEDVSYKDIPPLFRSGPGGRAKVGSYCVMDSVLVMDL 508
S_cerevisiae_DP_delta RE--YKLRSYTLNAVSAHFLGEQKEDVHYSIISDLQNGDSETRRRLAVYCLKDAYLPLRL 527
S_cerevisiae_DP_epsilon RDSYLPQGSQGLKAVTQSKLGYNPIELDPELMTPYAFEKP---QHLSEYSVSDAVATYYL 484
. . *.: : : :: : :. *.: *: *
V838
D831
W821
POL V
R839
Q827R
MHV68_DP TLSN------NKLLLKGVDLVRKTACKFVQSATSNVVSLILNDNDVRAAAQTMSNWG--- 854
HUMAN_DP_delta LLFSSRPDAHDRMDCKGLEAVRRDNCPLVANLVTASLRRLLIDRDPEGAVAHA------- 864
HUMAN_DP_epsilon FNED-----GSLAELKGFEVKRRGELQLIKIFQSSVFEAFLKGSTLEEVYGSVAKVADYW 1013
HSV-1_DP VIYG------GKMLIKGVDLVRKNNCAFINRTSRALVDLLFYDDTVSGAAAALAERP--- 994
HSV-2_DP VICG------GKMLIKGVDLVRKNNCAFINRTSRALVDLLFYDDTVSGAAAALAERP--- 999
VZV_DP VIYG------GKVLMKGVDLVRKNNCQFINDYARKLVELLLYDDTVSRAAAEASCVS--- 959
CMV_DP KVEG-----ASGLSMKGVDLVRKTACEFVKGVTRDVLSLLFEDREVSEAAVRLSRLS--- 1019
EBV_DP VLTD------GKTLMKGVELVRKTACKFVQTRCRRVLDLVLADARVKEAASLLSHRP--- 863
KSHV_DP VLSD------DKVLMKGVDLIRKTACRFVQEKSSQVLDLILREPSVKAAAKLISGQA--- 860
Herpesvirus_Saimiri_DP ILSN------DKLLMKGVDLVRKTACKFVQNTSSKILNLILKDPEVKAAAQLLSTKD--- 849
Rhesus_monkey_rhadinovirus_DP VLLN------DKMVMKGVDLIRKTACKFVQERCRAILDLVLHDPEVKAAARLLCKRP--- 863
S_cerevisiae_DP_delta LFWT-NPDKFDKLDQKGLASVRRDSCSLVSIVMNKVLKKILIERNVDGALAFV------- 870
S_cerevisiae_DP_epsilon FNED-----GSLAELKGFELKRRGELQLIKNFQSDIFKVFLEGDTLEGCYSAVASVCNRW 1026
. **. *: :: . .:
